# The structure of AcrIE4-F7 reveals a common strategy for dual CRISPR inhibition by targeting PAM recognition sites

**DOI:** 10.1093/nar/gkac096

**Published:** 2022-02-15

**Authors:** Sung-Hyun Hong, Gyujin Lee, Changkon Park, Jasung Koo, Eun-Hee Kim, Euiyoung Bae, Jeong-Yong Suh

**Affiliations:** Department of Agricultural Biotechnology, Seoul National University, Seoul 08826, Korea; Department of Agricultural Biotechnology, Seoul National University, Seoul 08826, Korea; Department of Agricultural Biotechnology, Seoul National University, Seoul 08826, Korea; Department of Agricultural Biotechnology, Seoul National University, Seoul 08826, Korea; Bio-Chemical Analysis Team, Korea Basic Science Institute, Ochang 28119, Korea; Department of Agricultural Biotechnology, Seoul National University, Seoul 08826, Korea; Research Institute of Agriculture and Life Sciences, Seoul National University, Seoul 08826, Korea; Department of Agricultural Biotechnology, Seoul National University, Seoul 08826, Korea; Research Institute of Agriculture and Life Sciences, Seoul National University, Seoul 08826, Korea

## Abstract

Bacteria and archaea use the CRISPR-Cas system to fend off invasions of bacteriophages and foreign plasmids. In response, bacteriophages encode anti-CRISPR (Acr) proteins that potently inhibit host Cas proteins to suppress CRISPR-mediated immunity. AcrIE4-F7, which was isolated from *Pseudomonas citronellolis*, is a fused form of AcrIE4 and AcrIF7 that inhibits both type I-E and type I-F CRISPR-Cas systems. Here, we determined the structure of AcrIE4-F7 and identified its Cas target proteins. The N-terminal AcrIE4 domain adopts a novel α-helical fold that targets the PAM interaction site of the type I-E Cas8e subunit. The C-terminal AcrIF7 domain exhibits an αβ fold like native AcrIF7, which disables target DNA recognition by the PAM interaction site in the type I-F Cas8f subunit. The two Acr domains are connected by a flexible linker that allows prompt docking onto their cognate Cas8 targets. Conserved negative charges in each Acr domain are required for interaction with their Cas8 targets. Our results illustrate a common mechanism by which AcrIE4-F7 inhibits divergent CRISPR-Cas types.

## INTRODUCTION

Bacteria and bacteriophages have co-evolved defense and counter-defense mechanisms that employ diverse molecular machinery. Among these, clustered regularly interspaced short palindromic repeats (CRISPRs) and CRISPR-associated (Cas) proteins constitute a bacterial defense system whereby invading genetic material can be recorded in the host genome to elicit a rapid immune response to subsequent infections (1). The components of the CRISPR-Cas system, which come in various shapes and sizes, can be broadly grouped into two classes. Class 1 (types I, III and IV) is characterized by multi-subunit effector complexes, whereas class 2 (types II, V and VI) comprises a single multi-domain protein for target interference. The type I CRISPR-Cas system is the most widely distributed in currently sequenced bacterial and archaeal genomes, and it is categorized into nine subtypes according to their signature *cas* genes (2,3). Type I Cas proteins associate with CRISPR RNA (crRNA) in a stoichiometric manner to form a CRISPR-associated complex for antiviral defense (Cascade) that binds target DNA and recruits dedicated nucleases for degradation (4).

To neutralize this bacterial defense system, bacteriophages express anti-CRISPR (Acr) proteins that potently inhibit CRISPR immunity. Acr proteins were first discovered in phages capable of disabling the type I-F CRISPR-Cas system of *Pseudomonas aeruginosa* UCBPP-PA14 (5). Efforts in functional assays and bioinformatic analyses lead to a growing number of Acr proteins that were often clustered in genomic sequences of phages and mobile genetic elements (6,7). AcrIE4 was identified in the *P. aeruginosa* phage D3112 inhibiting the type I-E CRISPR-Cas system of the strain SMC4386 through a functional assay, while AcrIF7 was identified using a bioinformatic approach in the *P. aeruginosa* PACS458 prophage (8,9). AcrIE4-F7, which features a concatenated sequence of AcrIE4 and AcrIF7, was later found in the mobile genetic element of *Pseudomonas citronellolis*. AcrIE4-F7 shows dual inhibition of the type I-E and type I-F CRISPR-Cas systems (10). It has been reported that *P. aeruginosa* possesses functional type I-F (11), I-E (12), I-C (13) and also IV-A (14) CRISPR-Cas systems.

AcrIF7 was recently reported to target the Cas8f subunit of the type I-F Cascade, blocking target DNA recognition by the protospacer adjacent motif (PAM) interaction site (15,16). Neither the structure of AcrIE4 nor its mechanism are known. Here, we solved the structure of AcrIE4-F7 using NMR spectroscopy, identifying its Cas targets and binding interfaces. AcrIE4-F7 adopts a novel α-helical fold in the N-terminal AcrIE4-like domain and a C-terminal αβ-fold that is homologous to the native AcrIF7 structure. Each domain binds the PAM interaction site of its cognate Cas8 subunit via conserved, charged residues, preventing access to target DNA. Our study demonstrates that PAM recognition sites are the primary targets of AcrIE4-F7 that counters divergent type I-E and type I-F CRISPR-Cas systems.

## MATERIALS AND METHODS

### Cloning, expression and purification

The synthetic AcrIE4-F7 gene was cloned into pET28a containing either N-terminal (His)_6_ or (His)_6_-maltose binding protein (MBP) tags with a tobacco etch virus (TEV) protease cleavage site. Mutant AcrIE4-F7 genes were generated using polymerase chain reaction (PCR) with mutagenic primers. Each construct was transformed into *Escherichia coli* BL21(DE3) cells, which were grown in LB medium at 37°C to an optical density at 600 nm of 0.6. Protein expression was induced by the addition of 0.5 mM isopropyl β-d-1-thiogalactopyranoside (IPTG) at 17°C for 16 h. The cells were then harvested by centrifugation and resuspended in lysis buffer (20 mM 3-(*N*-morpholino)propanesulfonic acid (MOPS), pH 7.0, 300 mM NaCl, 5 mM β-mercaptoethanol (BME), 10% (w/v) glycerol, 30 mM imidazole, 0.3 mM phenylmethylsulfonyl fluoride and 0.02% (w/v) Triton X-100). After sonication and centrifugation, the resulting supernatant was loaded onto a 5-mL HisTrap HP column (GE Healthcare) pre-equilibrated with binding buffer A (20 mM MOPS, pH 7.0, 300 mM NaCl, 5 mM BME, 10% (w/v) glycerol and 30 mM imidazole). After washing with the same buffer, the protein was eluted with a linear gradient of imidazole (up to 500 mM). The N-terminal (His)_6_-MBP tag was cleaved by TEV protease and separated using the 5-ml HisTrap HP column (GE Healthcare). AcrIE4-F7 was further purified by size-exclusion chromatography (SEC) using a HiLoad 16/60 Superdex 75 column (GE Healthcare) pre-equilibrated with SEC buffer A (20 mM 4-(2-hydroxyethyl)-1-piperazineethanesulfonic acid (HEPES), pH 7.0, 150 mM NaCl and 2 mM 1,4-dithiothreitol (DTT)).

The genetic fragments encoding the N-terminal and C-terminal domains of AcrIE4-F7 were amplified using PCR from its full-length gene and cloned into pET21a with a C-terminal (His)_6_ tag and the N-terminal (His)_6_-MBP tag with a TEV protease cleavage site, respectively. The resulting constructs were transformed into *E. coli* BL21(DE3) cells and expressed as described above for the full-length AcrIE4-F7. The proteins were loaded onto a 5-ml HisTrap HP column (GE Healthcare) pre-equilibrated with binding buffer A. After washing the column with the same buffer, the bound proteins were eluted with a linear gradient of imidazole (up to 500 mM). The (His)_6_-MBP tag of the C-terminal domain was cleaved by TEV protease and separated with the 5-ml HisTrap HP column (GE Healthcare). Finally, the proteins were purified by SEC using a HiLoad 16/60 Superdex 75 column (GE healthcare) pre-equilibrated with SEC buffer A.

To produce the Cas8f:Cas5f heterodimer, a subunit of the type I-F Cascade complex, synthetic Cas8f and Cas5f genes from *Xanthomonas albilineans* were cloned, respectively, into pET28a with an N-terminal (His)_6_-MBP tag and a TEV protease cleavage site and into pET21a without a tag. Both constructs were co-transformed into *E. coli* BL21(DE3) cells and co-expressed with 0.5 mM IPTG at 17°C for 16 h. The (His)_6_-MBP-tagged Cas8f:Cas5f heterodimer was loaded onto a 5-ml HisTrap HP column (GE Healthcare) pre-equilibrated with binding buffer B (20 mM tris(hydroxymethyl)aminomethane (Tris)–HCl, pH 7.5, 300 mM NaCl, 5 mM BME, 10% (w/v) glycerol and 30 mM imidazole). After washing the column with the same buffer, the protein sample was eluted with a linear gradient of imidazole (up to 500 mM). The N-terminal (His)_6_-MBP tag was cleaved by TEV protease and separated on a 5-ml HisTrap HP column (GE Healthcare). The Cas8f:Cas5f heterodimer was finally purified by SEC using a HiLoad 16/60 Superdex 200 column (GE Healthcare) pre-equilibrated with SEC buffer B (20 mM Tris–HCl, pH 7.5, 150 mM NaCl, 2 mM DTT and 5% (w/v) glycerol).

The genes of type I-E Cas proteins (i.e. Cas5e, Cas6e, Cas7e, Cas8e and Cas11) were amplified by PCR from *P. aeruginosa* PRD-10 and *E. coli* DH5α genomic DNAs. They were cloned into pET28a with an N-terminal (His)_6_-MBP tag and a TEV protease cleavage site. Mutant Cas8e genes were generated by site-directed mutagenesis using mutagenic PCR primers. The resulting wild-type (WT) and mutant constructs were transformed into *E. coli* BL21(DE3) cells. The type I-E Cas proteins were expressed individually as described above for the expression of AcrIE4-F7. The protein samples were purified without cleaving the (His)_6_-MBP tag because we found removal of the N-terminal tag destabilized the individual Cas proteins in our experimental conditions. The Cas proteins were then loaded onto a 5-mL HisTrap HP column (GE Healthcare) pre-equilibrated with binding buffer C (20 mM HEPES, pH 7.0, 500 mM NaCl, 5 mM BME, 20% (w/v) glycerol and 30 mM imidazole). After washing the column with the same buffer, the bound proteins were eluted with a linear gradient of imidazole (up to 500 mM). Finally, the proteins were purified by SEC using a HiLoad 16/60 Superdex 200 column (GE Healthcare) pre-equilibrated with SEC buffer A and 10% (w/v) glycerol.

### Analytical SEC

Analytical SEC was performed using a Superdex 200 10/300 GL column (GE Healthcare) pre-equilibrated with buffer (20 mM HEPES, pH 7.0, 150 mM NaCl, 2 mM DTT and 5% (w/v) glycerol). Proteins (20 μM each) were mixed and incubated at 4°C for 1 h, and then 700 μl of the mixture was loaded onto the SEC column at a flow rate of 0.5 ml/min. The eluted SEC fractions were analyzed by sodium dodecyl sulfate–polyacrylamide gel electrophoresis (SDS-PAGE) and visualized by Coomassie staining.

### Isothermal titration calorimetry (ITC)

ITC experiments were performed at 25°C using an iTC200 Calorimeter (Malvern). Samples in 200-μl cells were titrated with nineteen 2-μl injections. To analyze the binding of AcrIE4-F7 with the Cas8f:Cas5f heterodimer, AcrIE4-F7 (250 μM) was injected into a sample cell containing Cas8f:Cas5f (35 μM) in 20 mM Tris–HCl, pH 7.5, 150 mM NaCl, and 1 mM tris(2-carboxyethyl)phosphine (TCEP). For the interaction of AcrIE4-F7 (or its mutants) with Cas8e, we placed 20 μM of either AcrIE4-F7 or (His)_6_-MBP tagged Cas8e in the cell and titrated with 150–200 μM of the partner protein in 20 mM HEPES, pH 7.0, 150 mM NaCl, 1 mM TCEP and 5% (w/v) glycerol. The titrations were conducted in both directions, and the data were analyzed using the Origin software provided with the instrument.

### Multi-angle light scattering (MALS)

Static light scattering data were obtained using a Superdex 75 Increase 10*/*300 GL column (GE Healthcare) coupled with a miniDAWN (3-angle) light scattering detector (Wyatt Technology) and an Optilab T-rEX refractive index detector (Wyatt Technology). The column was equilibrated with 20 mM HEPES, pH 7.0 and 150 mM NaCl. Then, 100 μl of AcrIE4-F7 (150 μM) was loaded onto the column at a flow rate of 0.5 ml*/*min at 25°C. The results were analyzed using the ASTRA 8 software (Wyatt Technology).

### NMR spectroscopy

The NMR sample was prepared as 0.6 mM ^13^C,^15^N-labeled AcrIF4-F7 in 10 mM sodium phosphate, pH 7.0, 100 mM NaCl, 1 mM benzamidine and 10% (v*/*v) D_2_O. NMR spectra were obtained at 25°C on Bruker AVANCE III 800 MHz and AVANCE NEO 900 MHz spectrometers equipped with an *xyz*-shielded gradient triple resonance cryoprobe. NMR data were processed using the NMRPipe program (17) and analyzed using the PIPP*/*CAPP*/*STAPP (18) and NMRView (19) programs. Sequential backbone assignments were performed using 3D triple resonance through-bond scalar correlation experiments, which included HNCO, HN(CA)CO, HNCACB, CBCA(CO)NH and HBHA(CO)NH experiments. Side chain assignments were performed using HCCH-TOCSY, H(CCO)NH, and C(CO)NH experiments. Distance restraints were obtained using ^13^C-seperated NOESY and ^15^N-seperated NOESY experiments with a mixing time of 120 ms. {^1^H}–^15^N heteronuclear NOE measurements were acquired using 3 s of 120° ^1^H pulses separated by 5 ms intervals using a previously employed pulse program (20). Residual ^1^*D*_NH_ dipolar couplings were obtained by taking the difference in the ^1^*J*_NH_ splitting values measured in aligned (11.5 mg*/*ml of *pf1* phage, ASLA Biotech) and isotropic media using 2D in-phase*/*antiphase ^1^H–^15^N HSQC spectra.

### Structure calculation

Interproton distance restraints were derived from the NOE spectra and classified into distance ranges according to peak intensity. Backbone φ/ψ torsion angle restraints were derived from backbone chemical shifts using the program TALOS+ (21). Structures were calculated by simulated annealing in torsion angle space using the Xplor-NIH program (22). The target function for simulated annealing included covalent geometry, a quadratic van der Waals repulsion potential, square-well potentials for interproton distance and torsion angle restraints, hydrogen bonding, harmonic potentials for ^13^Cα*/*^13^Cβ chemical shift restraints (23), and a multidimensional torsion angle database potential of mean force (24).

### Multiple sequence alignment

Homologous sequences of AcrIE4-F7 were retrieved using the PSI-BLAST program (25), and redundant sequences (90% identity) were clustered using the CD-HIT program (26). The curated sequences were then aligned using the Clustal Omega program (27), and the multiple sequence alignment was analyzed and visualized using the Jalview program (28).

### Molecular docking

The model of the AcrIE4-F7:Cas8e complex was obtained using the HADDOCK 2.4 web server (29). We used the structural coordinates of the N-terminal domain of AcrIE4-F7 (from this study) and *P. aeruginosa* Cas8e (modeled from PDB code 5U07 and chain C; see the Results section). Key interfacial residues identified by SEC and ITC were used as ambiguous restraints for molecular docking. Active interfacial residues were defined as follows: Glu19, Tyr20, Asp22, Asp30 and Glu31 for the Acr proteins; Lys176, Lys183 and Lys357 for *P. aeruginosa* Cas8e. Passive interfacial residues were defined as those within 6.5 Å of the active residues. One thousand structures were generated via rigid body docking and energy minimization from random initial states, and the 200 lowest energy structures were selected for subsequent semi-flexible simulated annealing and explicit water refinement. The structure with the best HADDOCK score was displayed using the PyMOL software (The PyMOL Molecular Graphics System, Version 2.0 Schrödinger, LLC.).

## RESULTS

### The N- and C-terminal domains of AcrIE4-F7 bind to Cas8 subunits in type I-E and I-F CRISPR-Cas systems, respectively

**Figure 1.**
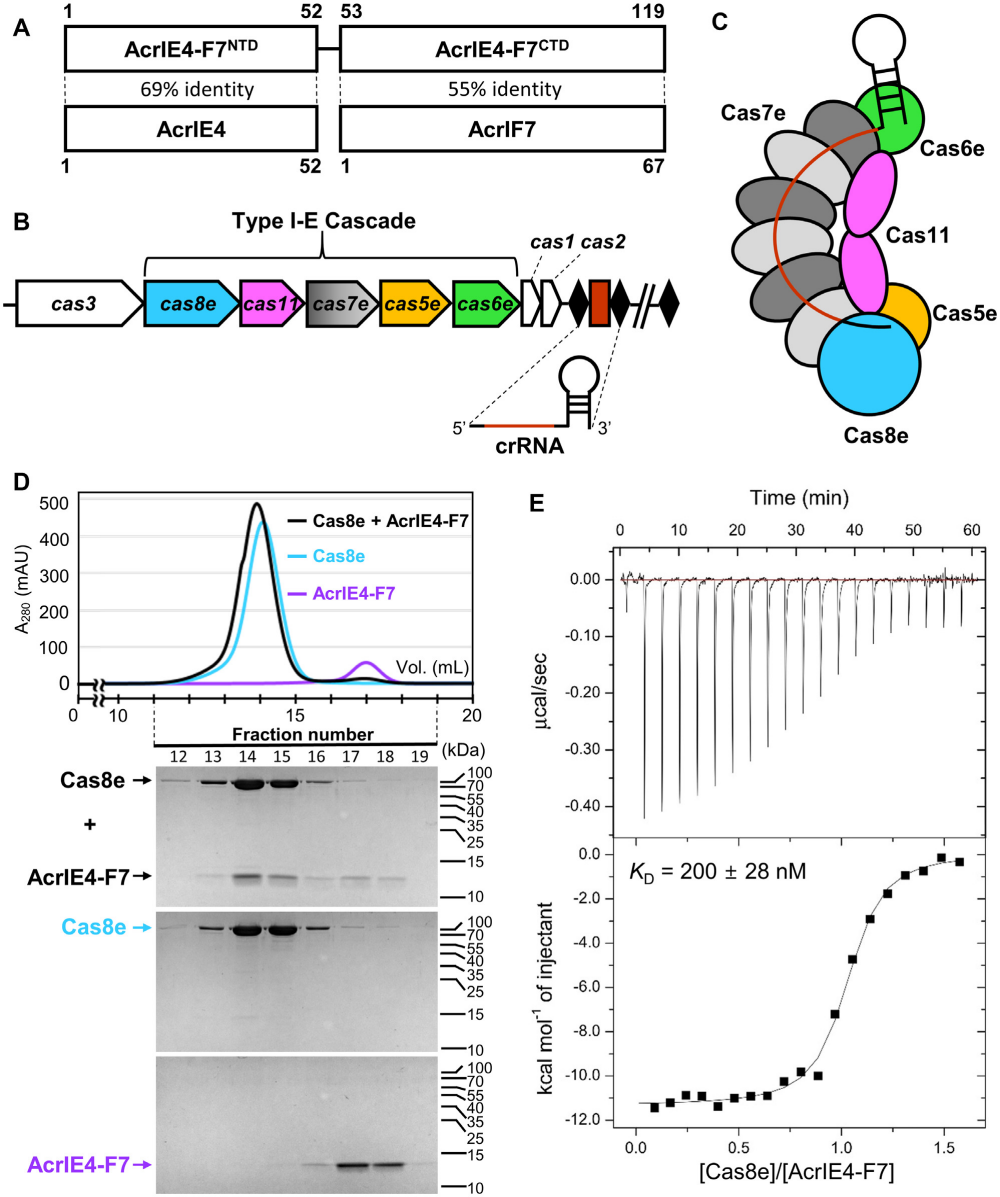
AcrIE4-F7 targets Cas8e in the type I-E CRISPR-Cas system of *P. aeruginosa*. (**A**) Domain organization of AcrIE4-F7. AcrIE4-F7^NTD^ and AcrIE4-F7^CTD^ each exhibit high sequence identity with native AcrIE4 and AcrIF7, respectively. (**B**) Schematic representation of the type I-E CRISPR-Cas locus. The black diamonds and the red rectangles indicate invariable repeats and variable phage-derived spacers, respectively. (**C**) The architecture of the type I-E Cascade complex. The complex displays a subunit stoichiometry of Cas8e_1_:Cas11_2_:Cas7e_6_:Cas5e_1_:Cas6e_1_:crRNA_1_. (**D**) Analytical SEC analysis for the interaction between AcrIE4-F7 and Cas8e. AcrIE4-F7 co-eluted with (His)_6_-MBP-tagged Cas8e. The elution fractions were analyzed by SDS-PAGE. Uncropped gel images are shown in Supplementary Figure S12. (**E**) ITC trace for the binding of (His)_6_-MBP-tagged Cas8e to AcrIE4-F7. The isotherm is representative of triplicate measurements and annotated with the average dissociation constant (*K*_D_) and standard error.

The C-terminal domain (AcrIE4-F7^CTD^; residues 53–119) of AcrIE4-F7 shares significant sequence similarity with native AcrIF7 (Figure 1A), whose structure and mechanism of inhibition have been investigated (15,16). Previously, we reported that AcrIF7 binds tightly to Cas8f, which itself forms a heterodimer with Cas5f to comprise the PAM-recognition ‘tail’ of the type I-F Cascade complex (Supplementary Figures S1A and B) (15). We asked whether AcrIE4-F7 interacts with the Cas8f:Cas5f subunit in a manner similar to the interaction of native AcrIF7. The binding experiments were performed with *X. albilineans* Cas8f:Cas5f, which we had previously used for analyzing the interaction with the native AcrIF7 (15). The sequence similarity is ∼50% between *X. albilineans* and *P. aeruginosa* Cas8f homologs, and the AcrIF7-interacting residues are completely conserved (15,16). The *X. albilineans* Cas8f:Cas5f bound tightly to type I-F Acr inhibitors such as AcrIF2 and AcrIF7 with submicromolar affinities (15,30). In an analytical SEC experiment, we found AcrIE4-F7 co-eluted with the Cas8f:Cas5f heterodimer (Supplementary Figure S1C). According to our ITC analysis, the equilibrium dissociation constant (*K*_D_) of AcrIE4-F7 with Cas8f:Cas5f falls around ∼26 nM (Supplementary Figure S1D), which is comparable to the *K*_D_ of ∼46 nM between AcrIF7 and Cas8f:Cas5f (15). Additional SEC and ITC experiments using truncated AcrIE4-F7 demonstrated that AcrIE4-F7^CTD^ was solely responsible for the tight association with Cas8f:Cas5f, yielding a *K*_D_ of ∼13 nM (Supplementary Figure S2). Thus, AcrIE4-F7^CTD^ is likely a structural and functional homolog of AcrIF7 capable of targeting the PAM interaction site of Cas8f to inhibit the type I-F CRISPR-Cas system.

Next, we sought to investigate the function of the N-terminal domain (AcrIE4-F7^NTD^; residues 1–52) of AcrIE4-F7, which exhibits a high level of sequence identity (69%) with native AcrIE4 (Figure 1A). AcrIE4 was identified as a potent inhibitor of the type I-E CRISPR-Cas system of *P. aeruginosa*, yet its target Cas component remains unclear. The identification of type I-E Acr targets has been difficult because the recombinant type I-E Cascade complex of *P. aeruginosa* exhibits poor expression and solubility (31). The type I-E Cascade is comprised of Cas8e, Cas11, Cas7e, Cas5e and Cas6e subunits. This resembles the composition of the type I-F Cascade, except for the addition of an extra Cas11 component (Figure 1B and C). The type I-E Cas proteins assemble into the Cascade with a stoichiometry of Cas8e_1_:Cas11_2_:Cas7e_6_:Cas5e_1_:Cas6e_1_ to process and associate with crRNA (Figure 1C). We learned that individual type I-E Cascade components can be expressed and purified using a solubility-enhancing N-terminal (His)_6_-MBP tag. Doing so, we successfully prepared each Cas subunit to test their *in vitro* binding to AcrIE4-F7. In our SEC analyses, although AcrIE4-F7 co-eluted with (His)_6_-MBP-tagged Cas8e (Figure 1D), no other Cascade subunits (Cas5e, Cas6e, Cas7e and Cas11) interacted with the Acr protein (Supplementary Figure S3). In an ITC experiment, we further observed a 1:1 binding between AcrIE4-F7 and Cas8e with a *K*_D_ value of ∼200 nM (Figure 1E). We note that Cas8e interacted exclusively with AcrIE4-F7^NTD^ (*K*_D_ ∼140 nM), but not with AcrIE4-F7^CTD^ in both the SEC and ITC experiments (Supplementary Figure S4). Together, our results demonstrate AcrIE4-F7^NTD^ and AcrIE4-F7^CTD^ bind to the Cas8e and Cas8f subunits, respectively, to mediate dual inhibition of CRISPR-Cas systems in *P. aeruginosa*.

AcrIE4-F7 did not interact with *E. coli* Cas8e in our SEC analysis (Supplementary Figure S5). This is consistent with previous plaque assay results showing AcrIE4 effectively suppressed the type I-E CRISPR-Cas system of *P. aeruginosa*, but not that of *E. coli* (8). The type I-E Cascade components of *P. aeruginosa* and *E. coli* are only distantly related, with pairwise sequence alignment identity scores for the individual Cas components varying between 7% and 34% (Supplementary Figure S6). *P. aeruginosa* Cas8e and its *E. coli* homolog preferentially recognize 5'-AAG and 5'-ATG PAM sequences, respectively, and Cas8e shows the lowest sequence homology of any of the subunits with an identity score of 7% (8,32). The specific Acr activity of AcrIE4 may be attributed to the divergent PAM interaction surfaces of the *P. aeruginosa* and *E. coli* Cas8e subunits (see below). Our observations collectively indicate that AcrIE4-F7^NTD^ targets the Cas8e subunit of the type I-E Cascade complex to accomplish CRISPR inhibition.

### AcrIE4-F7^NTD^ targets the PAM recognition site of Cas8e

Previous structural and mutational studies demonstrated that several type I-F Acr inhibitors target the PAM interaction site of the Cas8f subunit (33). These type I-F Acr proteins are highly acidic, with low theoretical pI values, and they compete with target DNAs for the Cas8f PAM binding site (34). In a previous study, we found positively charged Lys residues near the PAM recognition site of Cas8f to be essential for the interaction with the negatively charged AcrIF7 (15). Since AcrIE4-F7^NTD^ is also acidic (p*I* ∼4.2), we suspected that it too may function as a DNA mimic, interacting with the positively charged Cas8e PAM binding site. To test this hypothesis, we introduced charge-reversal mutations into Cas8e to determine the importance of positive charges in the interaction with AcrIE4-F7.

**Figure 2.**
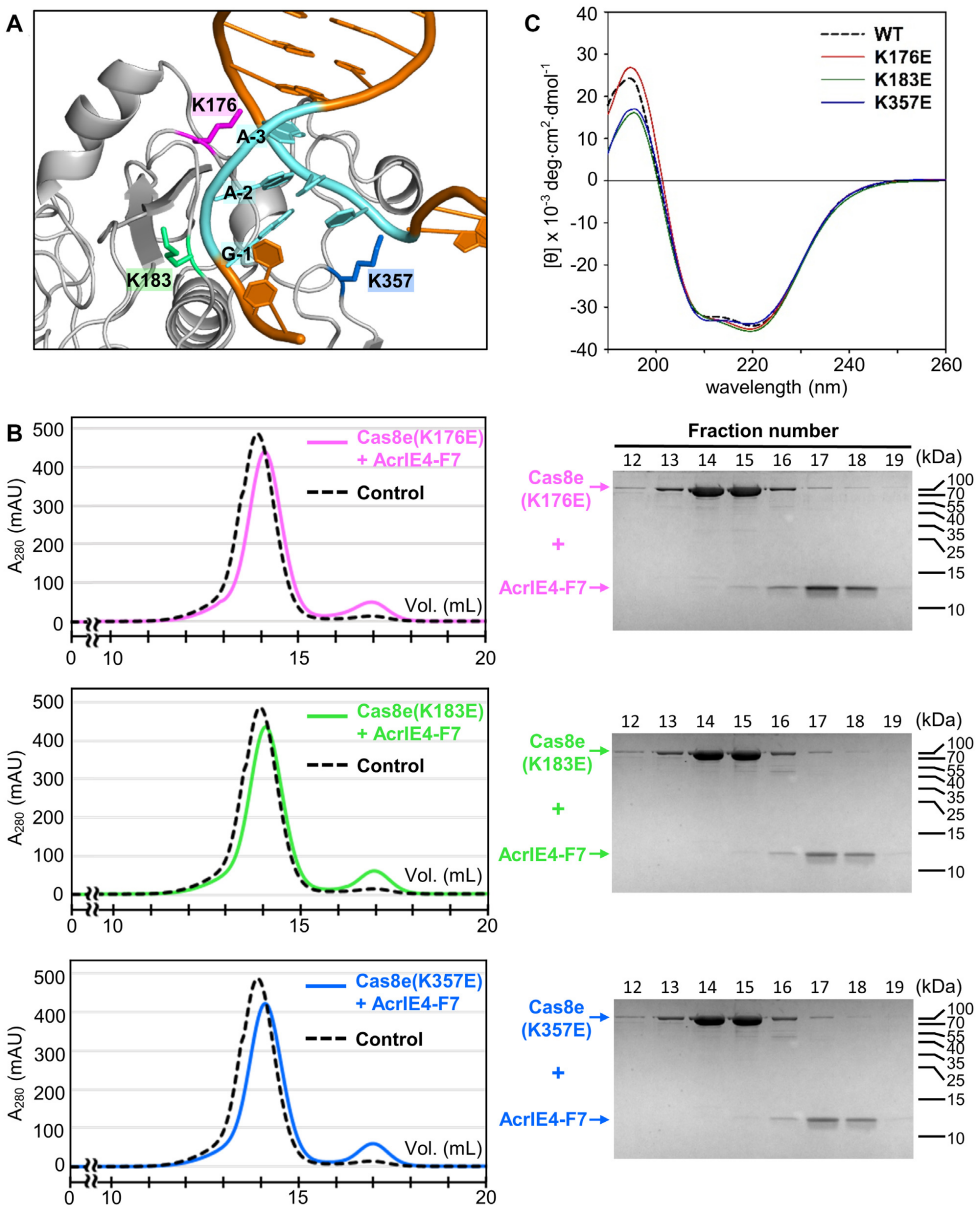
AcrIE4-F7 interacts with the putative PAM recognition site of Cas8e. (**A**) A model of *P. aeruginosa* Cas8e (*grey*) with a bound target DNA (*orange*) containing a 5'-AAG PAM sequence (*cyan*). The structure was modelled on the cryo-EM structure of the *T. fusca* Cascade:DNA complex (PDB code 5U07). Three Lys residues in the putative PAM recognition site of Cas8e—K176, K183 and K357—are shown in *pink*, *green*, and *blue*, respectively. (**B**) Analytical SEC analyses for the interaction between AcrIE4-F7 and Cas8e mutants. AcrIE4-F7 did not co-elute with any of the three Cas8e mutants, indicating that the charge-reversal mutations in the putative PAM recognition site of Cas8e disrupted the interaction with AcrIE4-F7. The SEC chromatogram (Figure 1D) for the binding to WT Cas8e is shown in dashed lines as a control for comparison. The elution fractions were analyzed by SDS-PAGE. Uncropped gel images are shown in Supplementary Figure S12. (**C**) CD spectra of WT and mutant Cas8e proteins. The three Cas8e mutants contain charge-reversal Lys-to-Glu substitutions in the putative PAM recognition site.

The structure of *P. aeruginosa* Cas8e is not currently available, but the structure of *Thermobifida fusca* type I-E Cascade has been determined by cryogenic electron microscopy (cryo-EM) (35). *T. fusca* Cas8e and *P. aeruginosa* Cas8e bear 24% sequence identity and recognize the same 5'-AAG PAM sequence (8,35), suggesting that they share conserved binding interfaces for PAM interaction. We modelled the *P. aeruginosa* Cas8e structure on the *T. fusca* Cas8e structure (PDB code 5U07) using the Phyre2 program (Figure 2A and Supplementary Figure S7) (36). *T. fusca* Cas8e harbors positively charged Arg208 and Arg386 in the Gly-rich loop and the Gln-wedge, respectively, and both residues are essential for PAM recognition (35). In the *T. fusca* Cas8e and *P. aeruginosa* Cas8e structural alignment, we found three Lys residues on *P. aeruginosa* Cas8e that lie within or adjacent to the Gly-loop (Lys176 and Lys183) or Gln-wedge (Lys357) (Figure 2A and Supplementary Figure S7). We generated *P. aeruginosa* Cas8e mutants by replacing each Lys residue one at a time with glutamate. In our SEC analyses, none of the three resulting Cas8e mutants interacted with AcrIE4-F7 (Figure 2B), indicating that these Lys residues are crucial for Acr binding. In ITC experiments, none of these mutants generated measurable isotherms upon titration with AcrIE4-F7. Since the Cas8e mutants exhibited similar CD spectra to that of WT Cas8e, it is unlikely that this lack of binding was caused by mutation-induced misfolding (Figure 2C). Together, our mutational analyses pinpoint the PAM recognition site of Cas8e as a putative binding interface for AcrIE4-F7. They suggest AcrIE4-F7^NTD^ mimics target DNA to compete for binding to the type I-E Cascade. In summary, two domains of AcrIE4-F7 employ a common strategy of blocking PAM recognition sites in their target Cas8 subunits to suppress the distinct type I-E and type I-F CRISPR-Cas systems.

### AcrIE4-F7 features compact individual folds linked in tandem

**Figure 3.**
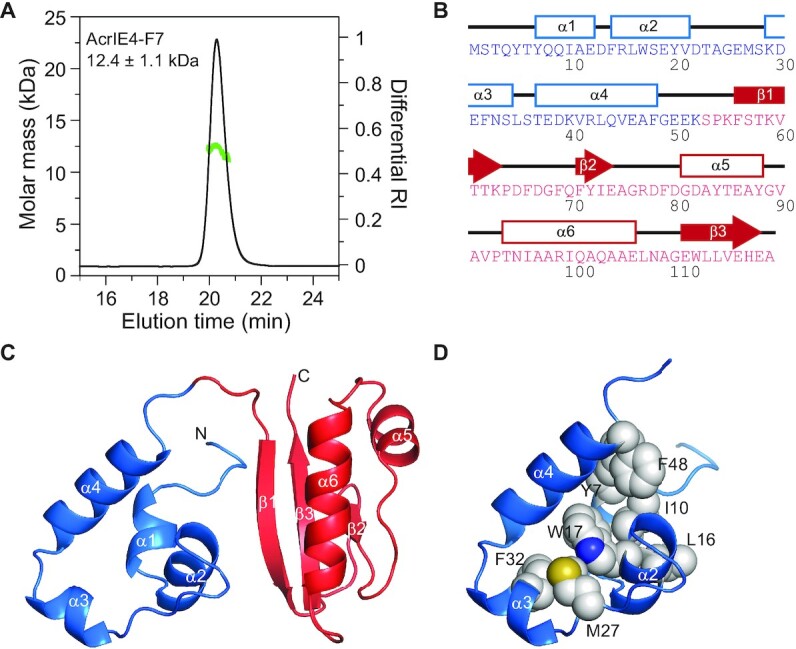
The structure of AcrIE4-F7. (**A**) SEC-MALS analysis of the molecular mass (*green*) and normalized refractive index (*black*) of AcrIE4-F7 indicates an AcrIE4-F7 monomer. (**B**) Schematic representation of secondary structural elements on the amino acid sequence of AcrIE4-F7. (**C**) Solution structure of AcrIE4-F7 as determined by NMR spectroscopy. The structures of AcrIE4-F7^NTD^ (*blue*) and AcrIE4-F7^CTD^ (*red*) are shown in a cartoon diagram. (**D**) Hydrophobic packing of nonpolar residues in AcrIE4-F7^NTD^. Aliphatic and aromatic side chains clustered in the hydrophobic core of AcrIE4-F7^NTD^ are annotated and shown in a space-filling model.

We used MALS and refractive index measurements to determine the oligomeric state of AcrIE4-F7. AcrIE4-F7 eluted as a monodisperse symmetric peak with an absolute molar mass of 12.4 ± 1.1 kDa (Figure 3A), which was close to the calculated molecular weight of 13 454.7 Da. This indicates AcrIE4-F7 appeared mainly in monomeric form in solution. We then employed a suite of triple-resonance heteronuclear correlation NMR spectroscopy techniques to assign the backbone and side chain ^1^H, ^15^N and ^13^C chemical shifts. We obtained distance restraints from 3D ^13^C-separated NOESY and ^15^N-separated NOESY experiments. Together, we employed 1625 NOE restraints, 234 dihedral angle restraints and 42 hydrogen bonding restraints to determine the solution structure of AcrIE4-F7 using the simulated annealing refinement protocol of the Xplor-NIH program (Table 1).

**Table 1.** Restraints and structural statistics for AcrIE4-F7

Experimental restraints	<SA>*
Nonredundant NOEs	1625
Intra-residue NOEs	769
Inter-residue NOEs	856
Sequential (| *i* – *j* | = 1)	366
Medium-range (1 < | *i* – *j* | ≤ 4)	207
Long-range (| *i* – *j* | > 4)	283
Dihedral angles, φ / ψ / χ	106/106/22
Hydrogen bonds	42
Total number of restraints	1901 (16.0 per residue)
Rms deviation from experimental restraints	
Distances (Å) (1625)	0.013 ± 0.001
Torsion angles (°) (234)	0.507 ± 0.068
Rms deviation from idealized covalent geometry	
Bonds (Å)	0.002 ± 0
Angles (°)	0.423 ± 0.005
Impropers (°)	0.276 ± 0.008
Coordinate precision (Å)^‡^	
Backbone, IE4 / IF7	0.93 ± 0.11/1.01 ± 0.13
Heavy atoms, IE4 / IF7	2.14 ± 0.11/2.09 ± 0.18
Ramachandran statistics (%)^‡^	
Favored regions	98 ± 1
Allowed regions	2 ± 1
Outliers	0

*For the ensemble of the final lowest-energy 20 simulated annealing structures.

^‡^The structured regions of AcrIE4-F7^NTD^ (residues 7–48) and AcrIE4-F7^CTD^ (residues 56–117) domains were fitted individually, excluding the flexible tail regions at the termini.

AcrIE4-F7 adopts four α-helices in the AcrIE4-F7^NTD^, as well as three antiparallel β-strands and two α-helices in the AcrIE4-F7^CTD^ (Figure 3B and C). AcrIE4-F7^NTD^ contains α1 (residues 7–12), α2 (residues 14–21), α3 (residues 29–34) and α4 (residues 37–48) helices, tightly packed with one another via hydrophobic interactions (Figure 3D). AcrIE4-F7^CTD^ folds like native AcrIF7 bound to the type I-F Cascade (PDB code 7JZX), a structure previously determined by cryo-EM (Supplementary Figure S8) (37). Apart from the flexible β1–β2 loop region, their backbone folds superimpose nicely against each other, yielding a root-mean-square deviation of 1.4 Å for 60 Cα atom positions. A DALI search for structural homologs of AcrIE4-F7 with *Z*-scores larger than 3.0 returned the free AcrIF7 structure (PDB code 6M3N) and the AcrIF7:Cascade complex structure (PDB code 7JZX) (15,37). A similar search using a truncated AcrIE4-F7^NTD^ coordinate, however, failed to find any similar structures, suggesting that the helical topology of AcrIE4-F7^NTD^ is unique in the public database.

### AcrIE4-F7 domains are tethered by a flexible linker

**Figure 4.**
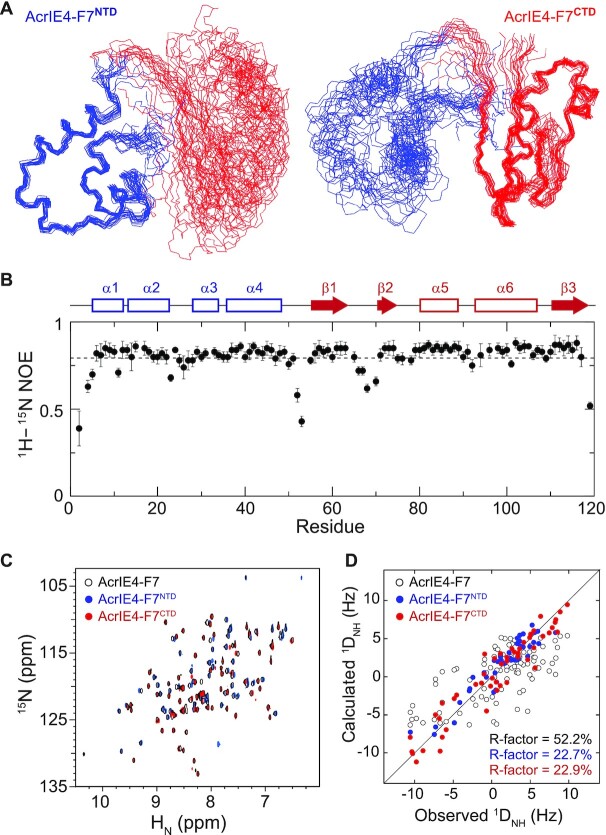
Inter-domain motion of AcrIE4-F7. (**A**) Superposition of the backbone atoms of the final 20 simulated annealing structures of AcrIE4-F7. These structures are best-fit superposed on secondary structures of AcrIE4-F7^NTD^ (*left*) and AcrIE4-F7^CTD^ (*right*). (**B**) {^1^H}–^15^N heteronuclear NOE data as a function of residue number. The secondary structures of AcrIE4-F7 are indicated above the graph. (**C**) Superimposed 2D ^1^H–^15^N HSQC spectra of ^15^N-labeled AcrIE4-F7 (*black*), truncated AcrIE4 (*blue*), and AcrIF7 (*red*) domains. (**D**) Comparison of the observed ^1^D_NH_ residual dipolar couplings for the backbone amide resonances of AcrIE4-F7 with those calculated from the atomic coordinates of the solution structure. RDC R-factors derived from individual fitting with AcrIE4 (*blue*), AcrIF7 (*red*) domain, or simultaneous fitting with both domains (*black*). Calculations for the fitting with AcrIE4-F7^NTD^, AcrIE4-F7^CTD^, or the whole AcrIE4-F7 over all 20 structures in the ensemble produced *R*-factors of (22.7 ± 2.2)%, (24.3 ± 2.6)%, and (48.7 ± 4.0)%, respectively.

The secondary structures of AcrIE4-F7^NTD^ and AcrIE4-F7^CTD^ alone are well-defined in the 20 lowest-energy structures, but the two domains do not align simultaneously over the entire length of AcrIE4-F7, suggesting the presence of inter-domain motion (Figure 4A). We asked whether this domain motion might be an artifact arising from insufficient experimental distance restraints between the two domains. First, we measured the {^1^H}–^15^N heteronuclear NOE of the backbone amide resonances in AcrIE4-F7 to identify any flexible segments. Large NOE values (>0.8) prevailed throughout the secondary structure, indicating that each individual domain maintained rigid folds (Figure 4B). In contrast, the linker connecting AcrIE4-F7^NTD^ and AcrIE4-F7^CTD^ exhibited significant mobility (Figure 4B). Apart from the terminal tails, linker residues Lys52 and Ser53 showed the lowest NOE values at 0.58 and 0.43, respectively. This suggests that these two residues form a flexible linker between the two domains. Second, we found that the backbone amide resonances of truncated AcrIE4-F7^NTD^ and AcrIE4-F7^CTD^ constructs could be superimposed with those of intact AcrIE4-F7 in the HSQC spectra, except for the linker region (Figure 4C). The absence of chemical shift perturbations indicates that the linked domains of AcrIE4-F7 do not interact with one another in any specific way. Last, we obtained residual dipolar couplings (RDCs) of the backbone amides in *pf1* phage alignment medium to determine whether the two domains exhibit correlated rigid-body motion in solution. The experimental RDCs agreed well with the atomic coordinates of AcrIE4-F7 after fitting them to the individual domains of AcrIE4-F7^NTD^ or AcrIE4-F7^CTD^ (Figure 4D). The simultaneous fit of both domains, however, produced poor agreement with any single structure of the conformational ensemble, illustrating that the domains exhibit uncorrelated motion in dynamic equilibrium. (Figure 4D). Taken together, our results demonstrate that AcrIE4-F7 explores multiple conformational states with varying orientations between its N-terminal and C-terminal domains.

### Binding interface of AcrIE4-F7^NTD^ for Cas8e

**Figure 5.**
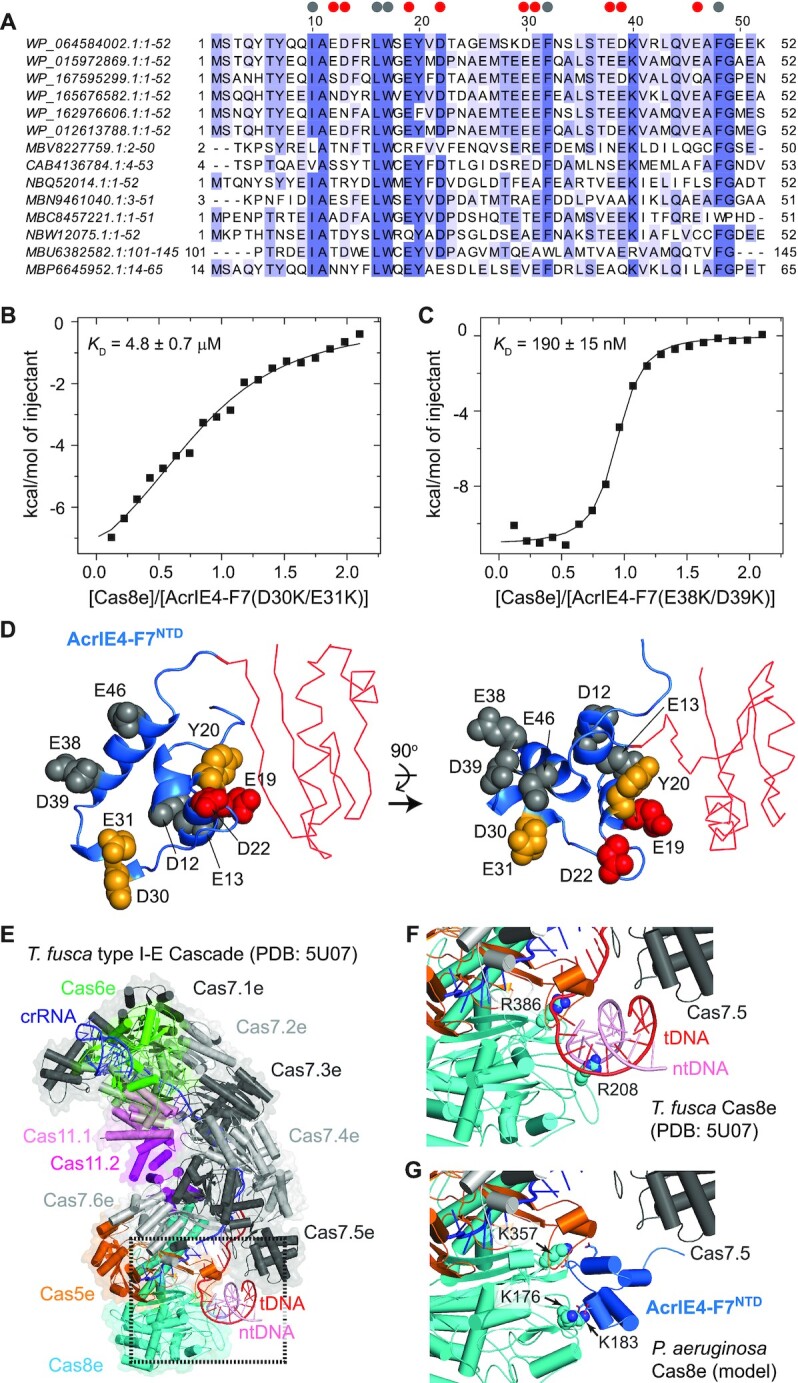
Interaction of AcrIE4-F7^NTD^ and Cas8e. (**A**) Multiple sequence alignment of AcrIE4-F7^NTD^. The sequence numbering above the sequence is based on the AcrIE4-F7 sequence at the top, and negatively charged residues for mutagenesis and conserved nonpolar residues are indicated with *red* and *gray* circles, respectively. The sequences are preceded by the GenBank ID, and the names of source organisms are as follows: WP_064584002.1 (*P. citronellolis*; this study), WP_015972869.1 (*P. aeruginosa*), WP_167595299.1 (*Chromobacterium subtsugae*), WP_165676582.1 (*Pseudomonas otitidis*), WP_162976606 (*P. aeruginosa*), WP_012613788.1 (*P. aeruginosa*), MBV8227759.1 (Verrucomicrobia bacterium, rhizosphere metagenome), CAB4136784.1 (uncultured *Caudovirales* phage), NBQ52014.1 (Proteobacteria bacterium, freshwater metagenome), MBN9461040.1 (Burkholderiales bacterium, bioreactor metagenome), MBC8457221.1 (Deltaproteobacteria bacterium, marine metagenome), NBW12075.1 (*Caulobacteraceae* bacterium, freshwater metagenome), MBU6382582.1 (Proteobacteria bacterium, mine drainage metagenome), MBP6645952.1 (*Burkholderiaceae* bacterium, wastewater metagenome). Integrated heats for the binding of (**B**) AcrIE4-F7(D30K/E31K) and (**C**) AcrIE4-F7(E38K/D39K) mutants to Cas8e in ITC experiments. The isotherms are representative of triplicate measurements and annotated with their average dissociation constants (*K*_D_) and standard errors. (**D**) The AcrIE4-F7 structure as a cartoon (*blue*; AcrIE4-F7^NTD^) and a Cα trace (*red*; AcrIE4-F7^CTD^) diagram. Side chains are shown in a space-filling model and color-coded by their contributions to Cas8e binding: large (*red*), medium (*orange*), and negligible (gray). The structure is viewed from the front (*left* panel) and from the top (*right* panel). (**E**) Cartoon diagram of the *T. fusca* type I-E Cascade complex bound to crRNA and target dsDNA (PDB code 5U07). (**F**) A close-up view of the PAM interaction site of Cas8e from the dotted box in panel E. (**G**) A model structure of AcrIE4-F7^NTD^ bound to *P. aeruginosa* Cas8e within the *T. fusca* Cascade complex. ntDNA, non-target DNA strand; tDNA, target DNA strand.

A PSI-BLAST search for AcrIE4-F7 returned homologs of the AcrIE4 and AcrIF7 domains, but it did not find any homolog for the full-length linked AcrIE4-F7. AcrIE4 homologs were identified in gamma-proteobacteria (e.g. *Pseudomonas* species), while AcrIF7 homologs were distributed among both gamma- and beta-proteobacteria (e.g. *Janthinobacterium* species). On a multiple sequence alignment of AcrIE4-F7^NTD^, we identified both charged and hydrophobic residues conserved across homologs (Figure 5A). A few aliphatic (Ile10 and Leu16) and aromatic (Trp17, Phe32 and Phe48) residues in the hydrophobic core were highly conserved, suggesting their importance in proper folding.

Since AcrIE4-F7^NTD^ interacted with positive charges in Cas8e, we mutated negatively charged residues and monitored the interaction of the resulting AcrIE4-F7 mutants with Cas8e via analytical SEC and ITC experiments. Mutations in the α2 helix (E19K/D22K) drastically impaired the interaction between AcrIE4-F7 and Cas8e, completely preventing binding of the mutant to Cas8e (Table 2 and Supplementary Figure S9). We further confirmed that both E19K and D22K were crucial for the interaction with Cas8e, since individual mutations of either residue abolished Cas8e binding (Table 2). Mutations in the α3 helix (D30K/E31K) also significantly affected Cas8e binding, producing a 24-fold reduction in binding affinity (Figure 5B and Table 2). On the other hand, mutations in the α1 helix (E12K/D13K) and in the α4 helix (E38K/D39K and E46K) had only modest or no effect on binding affinity. While the E12K/D13K and E46K mutants showed a two-fold reduction in Cas8e binding (Table 2 and Supplementary Figures S10A and C), the E38K/D39K mutation did not affect binding affinity at all (Figure 5C and Table 2). Last, we found that a Y20A mutation in the α2 helix reduced binding affinity by ∼18-fold (Table 2 and Supplementary Figure S10B). This supports the hypothesis that the α2 helix serves as the main binding interface for Cas8e. We note that the Tyr20 position is also highly conserved in the sequence alignment across AcrIE4-F7^NTD^ homologs (Figure 5A). We confirmed that all the mutants used in this study maintained their secondary structures, since the CD spectra of the mutants remained unchanged from that of WT AcrIE4-F7 (Supplementary Figure S11).

**Table 2.** Thermodynamic parameters from ITC experiments between Cas8e and AcrIE4-F7 mutants^a^

**AcrIE4-F7**	**Cas8e**	***K*_D_ (nM)**	**N**	**Δ*G* (kcal/mol)**	**Δ*H* (kcal/mol)**	**−*T*Δ*S* (kcal/mol)**
WT	WT	200 ± 28	1.0 ± 0.0	−9.2 ± 0.1	−14.7 ± 1.4	5.5 ± 1.5
E12K/D13K	WT	400 ± 120	0.8 ± 0.0	−8.8 ± 0.2	−22.8 ± 1.8	14.0 ± 1.9
E19K/D22K	WT	N.B.^b^	-	-	-	-
E19K	WT	N.B.	-	-	-	-
D22K	WT	N.B.	-	-	-	-
Y20A	WT	3500 ± 1300	1.1 ± 0.2	−7.6 ± 0.3	−5.6 ± 1.5	−2.0 ± 1.5
D30K/E31K	WT	4800 ± 690	0.8 ± 0.1	−7.3 ± 0.1	−11.1 ± 1.8	3.8 ± 1.7
E38K/D39K	WT	190 ± 15	0.9 ± 0.1	−9.2 ± 0.0	−12.8 ± 2.2	3.6 ± 2.2
E46K	WT	430 ± 30	1.0 ± 0.1	−8.7 ± 0.0	−11.7 ± 1.5	3.0 ± 1.5
AcrIE4-F7^NTD^	WT	140 ± 40	0.8 ± 0.1	−9.4 ± 0.2	−18.1 ± 1.7	8.7 ± 1.5

^a^ITC experiments were performed in triplicate, and their thermodynamic parameters are reported as average values with standard errors.

^b^No binding: Integrated heats from the measurement were insufficient to constrain the least squares fit derived from a single-site binding model for the titration.

We found the key residues of AcrIE4-F7^NTD^ for Cas8e binding clustered to form a contiguous binding interface that was not occluded by the linked C-terminal AcrIF7 domain (Figure 5D). We carried out molecular docking of AcrIE4-F7 onto *P. aeruginosa* Cas8e using the HADDOCK program based on the interfacial residues of AcrIE4-F7 and Cas8e. The type I-E Cascade assumes a sea horse-like architecture in which six Cas7e subunits assemble along a crRNA to form a backbone and Cas8e and Cas5e join to form the tail (Figure 5E). Cas8e recognizes the PAM site of substrate DNA via positively charged residues, which leads to the strand invasion by crRNA (Figure 5F). We explored the complex structure of AcrIE4-F7^NTD^ and Cas8e in the context of type I-E Cascade assembly by replacing the coordinates of *T. fusca* Cas8e with those of *P. aeruginosa* Cas8e (Figure 5G). Our model visualizes that AcrIE4-F7^NTD^ blocks the PAM interaction site of Cas8e to compete with target DNA binding (Figure 5F and G). We note that the molecular docking was performed between AcrIE4-F7^NTD^ and *P. aeruginosa* Cas8e, and then interpreted in the context of full-length AcrIE4-F7 and the type I-E Cascade assembly. This approach can avoid a potential pitfall of a rigid-body docking that does not take account of the torsional flexibility of the linker. When we superimposed the full-length AcrIE4-F7 structure on AcrIE4-F7^NTD^ in the complex model, some of the conformers showed partial overlaps between with AcrIE4-F7^CTD^ and Cascade subunits (Cas5e or Cas7e), but the steric clash vanished upon small rotations at the linker conformation. In sum, the conformational freedom at the linker likely allows facile domain reorientations that ease the access of AcrIE4-F7 to the cognate site on Cas8e.

### The binding interface between AcrIE4-F7^CTD^ and Cas8f

**Figure 6.**
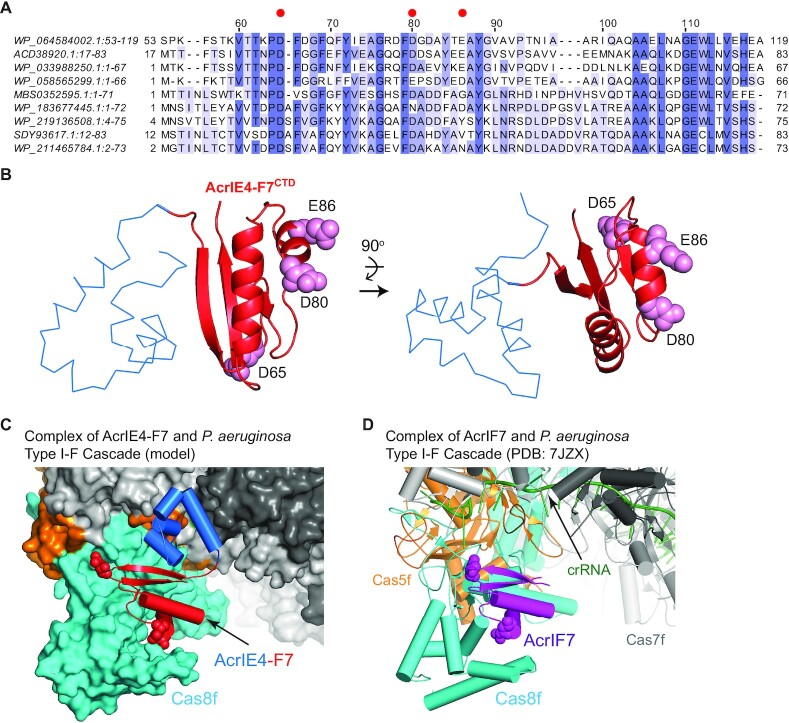
The interaction of AcrIE4-F7^CTD^:Cas8f. (**A**) Multiple sequence alignment of AcrIE4-F7^CTD^. The sequence numbering above the sequence is based on the AcrIE4-F7 sequence at the top, and conserved negatively charged residues at the binding interface for Cas8f are indicated with *red* circles. The sequences are preceded by the GenBank ID, and the names of source organisms are as follows: WP_064584002.1 (*P. citronellolis*; this study), ACD38920.1 (*P. aeruginosa*), WP_033988250.1 (*P. aeruginosa*), WP_058565299.1 (*P. aeruginosa*), MBS0352595.1 (Proteobacteria bacterium, activated sludge metagenome), WP_183677445.1 (unclassified *Janthinobacterium*), WP_219136508.1 (*Janthinobacterium* sp. UMAB-60), SDY93617.1:13–83 (*Collimonas* sp. OK242), WP_211465784.1 (*Collimonas silvisoli*) (**B**) The structure of AcrIE4-F7 shown as a Cα trace (*blue*; AcrIE4-F7^NTD^) and a cartoon (*red*; AcrIE4-F7^CTD^) diagram in front (*left* panel) and top (*right* panel) views. Interfacial side chains are shown as a space-filling model in *pink*. (**C**) A model structure of AcrIE4-F7 (*blue* and *red*) bound to the type I-F Cascade. (**D**) The cryo-EM structure of AcrIF7 (*purple*) bound to the type I-F Cascade (PDB code 7JZX) of *P. aeruginosa*. The interfacial residues of ArcIE4-F7 are shown as a space-filling model. The Cascade components are colored as follows: Cas8f (*cyan*), Cas5f (*orange*), Cas7f (*light* and *dark gray*), and crRNA (*dark green*).

A multiple sequence alignment of the AcrIF7 domain revealed a strong conservation of the key interfacial residues for Cas8f (Figure 6A). Specifically, Asp65, Asp80 and Glu86 of AcrIE4-F7^CTD^ (Asp13, Asp28 and Glu34 in the native AcrIF7 sequence) were mostly conserved and exhibited the largest impact on Cas8f binding when mutated (15). These residues mimic the phosphate group of the PAM sequence to compete with target DNA binding to the type I-F Cascade (16). The key residues for Cas8f binding were located on the opposite side of the N-terminal AcrIE4 domain (Figure 6B). When we superimposed the AcrIE4-F7^CTD^ upon AcrIF7 in complex with the type I-F Cascade of *P. aeruginosa* (PDB code 7JZX), AcrIE4-F7 docked snugly to its target Cas8f without steric collision of AcrIE4-F7^NTD^ and the other Cas subunits in the Cascade (Figure 6C and D).

## DISCUSSION

PAM recognition is the key step that primes the CRISPR-Cas system to find and cleave target nucleic acids. Mutations in PAM sequences are frequently observed in phages that have escaped the CRISPR surveillance of their host bacteria, highlighting the importance of PAM interactions in the defense mechanism (38,39). Structural investigations of type I-F Acr proteins revealed that AcrIF2, AcrIF6, AcrIF7 and AcrIF10 can interfere with PAM recognition of their target Cas8f (16,40–42). Structural and mechanistic studies of type I-E Acr proteins, in contrast, have been limited, with only the individual structures of AcrIE1 and AcrIE2 available to date. AcrIE1 was found to interact with Cas3, suggesting that it may interfere with Cas3 recruitment to the Cascade in a mechanism similar to that of AcrIF3 (43,44). Although the Cas target of AcrIE2 remains unknown, AcrIE2 reportedly failed to block target DNA binding to the Cascade (31). Thus, our study suggests AcrIE4 employs a mechanism previously unknown among type I-E Acrs, achieving CRISPR inhibition by blocking the PAM interaction site. Given that Cas8 homologs are ubiquitous among the type I CRISPR-Cas systems, we speculate that this Cas8 targeting inhibitory mechanism will also be identified in other type I Acr families (2).

To identify the type I-E Cas target of AcrIE4-F7, we used individual Cascade components expressed and purified with N-terminal (His)_6_-MBP tags. In contrast with the *E. coli* Cascade, the *P. aeruginosa* Cascade is difficult to obtain as a recombinant protein complex due to its poor expression and solubility (31). Our approach has the following potential limitations: (i) The N-terminal MBP tag may occlude potential binding interfaces for Acr if the interaction takes place near the N-terminus; (ii) the Acr binding interface may comprise multiple Cascade components and (iii) the individual Cas proteins may not fold correctly without other interacting Cascade subunits. Notwithstanding, we were able to show that AcrIE4-F7 binds only to the Cas8e subunit of the type I-E Cascade components with submicromolar affinity, suggesting Cas8e is the main target for the Acr inhibitor. Our mutational and modeling analyses indicate that the Acr-interacting Cas8e residues are not close to its N-terminus. We cannot, however, rule out the possibility that the presence of other subunits in the Cascade may enhance AcrIE4-F7 binding affinity. In type I-F systems, several Cas8f-interacting Acr proteins (e.g. AcrIF4, AcrIF6 and AcrIF10) make additional contacts with neighboring Cascade subunits (16,41,42). Also, AcrIF2 binds more tightly to the Cas8f:Cas5f heterodimer than to the Cas8f subunit alone (30). We attempted to obtain a stable Cas8e:Cas5e heterodimer to measure its binding affinity to AcrIE4-F7, but we were unable to produce a soluble complex using our co-expression system.

Dual inhibition of type I-E and I-F CRISPR-Cas systems may be beneficial for phage survival, given that these two CRISPR types are the most common, often co-existing in the sequenced genomes of *P. aeruginosa* (13,45). For example, phylogenetic studies of CRISPR-Cas systems in *P. aeruginosa* revealed that 12 out of 672 genomes contained both type I-E and I-F CRISPR-Cas systems (13). Not surprisingly, Acrs occasionally appear to inhibit different CRISPR-Cas types, such that AcrIF6, AcrIF18.1, AcrIF18.2, and AcrIF22 simultaneously inhibit type I-E and I-F CRISPR-Cas systems (7,9). AcrIF6 adopts a compact α-helical fold and binds to Cas8f and Cas7.6f of the type I-F Cascade, but its mechanism of dual inhibition remains unknown (42). AcrIF18.1, AcrIF18.2 and AcrIF22 are small proteins (7.7–9.8 kDa) for which we lack structural information, yet sequence alignments suggest they are not multi-domain proteins. Thus, AcrIE4-F7 is unique in that two functionally independent Acr proteins are fused to form a dual CRISPR inhibitor. It is plausible that clustered *acr* genes in the anti-defense island merged to produce a multitarget inhibitor on an evolutionary time scale. Previously, *acrIE4* was found close to *acrIF2*, *acrIF3* and *acrIF5* in the Acr locus of prophages in *P. aeruginosa* (8). Despite looking, we were unable to find any evidence of the co-location of *acrIE4* and *acrIF7* loci across the archived microbial and phage genome sequence databases. Nevertheless, because phage genome sequences are underrepresented in the existing databases, further effort spent on metagenomic sequencing may clarify the evolutionary origin of AcrIE4-F7.

As natural CRISPR inhibitors, Acr proteins show great potential in gene editing and transcriptional control applications (46). There are ongoing efforts to engineer Acrs for improved inhibition potency and selectivity (47). Tethering Acr proteins that bind to the Cas target at non-overlapping interfaces may help enhance inhibition potency. For example, AcrIF1 that binds to the type I-F Cascade in tandem may exhibit a higher affinity to its target by introducing a linker. As a promising strategy for regulating Cas targets, the combination and concatenation of Acrs warrants future experimental effort.

## DATA AVAILABILITY

The atomic coordinates of the AcrIE4-F7 solution structure and NMR restraints have been deposited in the Protein Data Bank (PDB code 7VZM) and the Biological Magnetic Resonance Bank (accession code 36454), respectively.

## Supplementary Material

gkac096_Supplemental_File
