# Supplementary material for: The structure of AcrIE4-F7 reveals a common strategy for dual CRISPR inhibition by targeting PAM recognition sites
Source: Nucleic Acids Res. 2022 Feb 15;50(4):2363–76. doi: 10.1093/nar/gkac096 (PMC8887544; doi:10.1093/nar/gkac096)

Supplementary Information

The structure of AcrIE4-F7 reveals a common strategy for dual CRISPR inhibition by targeting PAM recognition sites

Sung-Hyun Hong, Gyujin Lee et al.

Figure S1. AcrIE4-F7 binds to the Cas8f:Cas5f heterodimer. (A) Schematic representation of the type I-F CRISPR-Cas locus. The black diamonds and the red rectangles indicate invariable repeats and variable phage-derived spacers, respectively. (B) The architecture of the type I-F Cascade complex. The type I-F CRISPR surveillance complex displays a subunit stoichiometry of $Cas8f_1$:$Cas7f_6$:$Cas5f_1$:$Cas6f_1$:$crRNA_1$. (C) Analytical SEC analysis for the interaction between AcrIE4-F7 and the Cas8f:Cas5f subcomplex. AcrIE4-F7 co-eluted with the Cas8f:Cas5f heterodimer. The elution fractions were analyzed by SDS-PAGE. Uncropped gel images are shown in Figure S12. (D) ITC trace for the binding of AcrIE4-F7 to the Cas8f:Cas5f heterodimer. AcrIE4-F7 was injected consecutively to the Cas8f-Cas5f heterodimer. The experimentally determined dissociation constant (K_D) value is indicated.

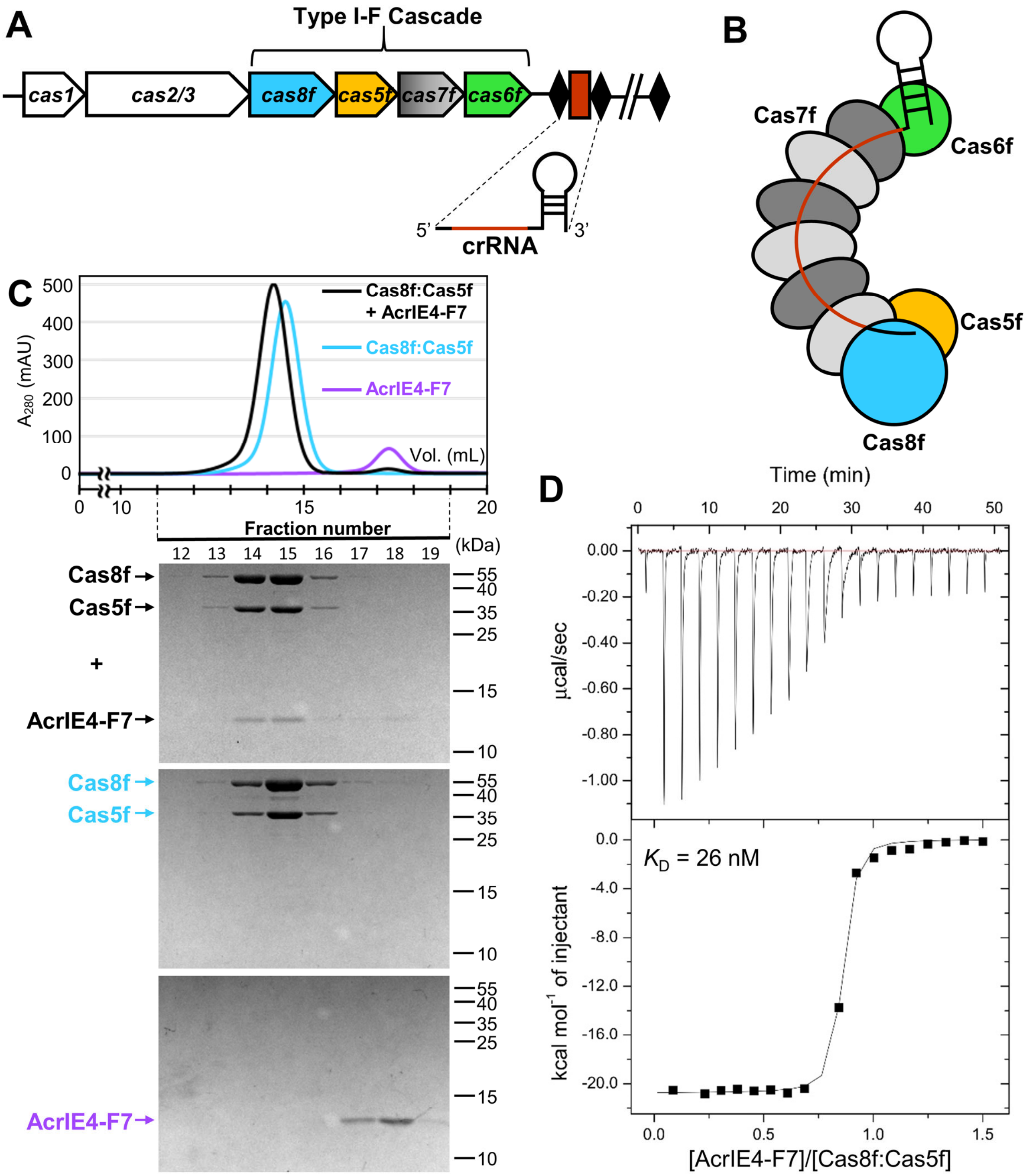

Figure S2. The C-terminal domain of AcrIE4-F7 is responsible for its interaction with Cas8f:Cas5f. (A) Analytical SEC analyses for the interactions between Cas8f:Cas5f and the AcrIE4-F7 N- and C-terminal domains. The Cas8f:Cas5f heterodimer co-eluted with AcrIE4-F7^{CTD} (*right*), but not with AcrIE4-F7^{NTD} (*left*). The elution fractions were analyzed by SDS-PAGE. Uncropped gel images are shown in Figure S12. (B) ITC trace for the binding of AcrIE4-F7^{CTD} to the Cas8f:Cas5f heterodimer. The experimentally determined dissociation constant (K_D) value is indicated.

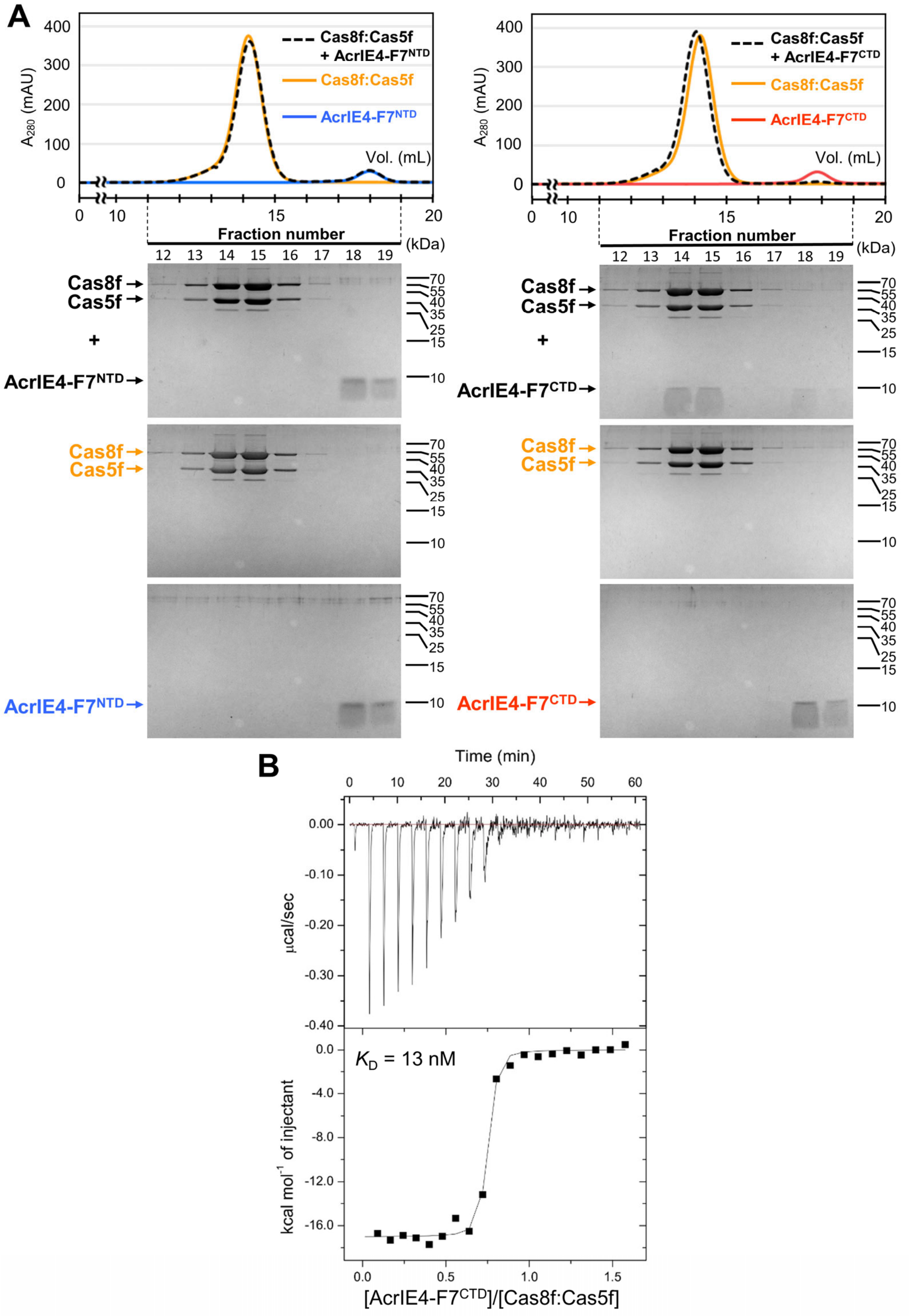

Figure S3. AcrIE4-F7 does not interact with other type I-E Cas proteins (i.e., Cas5e, Cas6e, Cas7e, or Cas11) that comprise the *P. aeruginosa* Cascade complex. Analytical SEC analyses for the interaction between AcrIE4-F7 and Cas5e (A), Cas6e (B), Cas7e (C), or Cas11 (D). AcrIE4-F7 did not co-elute with any of the other type I-E Cascade components. The elution fractions were analyzed by SDS-PAGE. Uncropped gel images are shown in Figure S12.

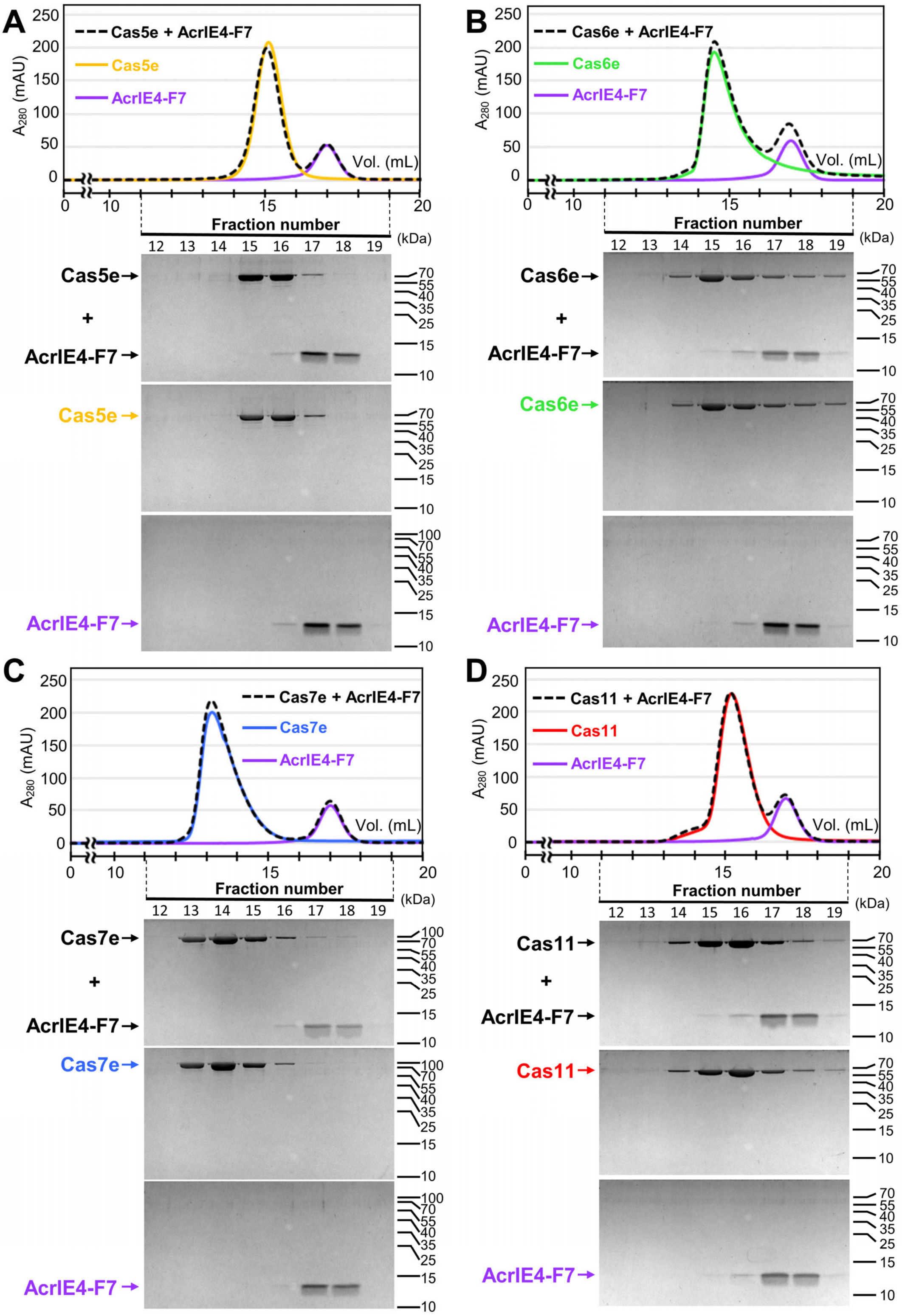

Figure S4. Cas8e interacts with the N-terminal domain of AcrIE4-F7, but not with its C-terminal domain. (A) Analytical SEC analyses for the interactions between Cas8e and the AcrIE4-F7 N- and C-terminal domains. Cas8e co-eluted with AcrIE4-F7^{NTD} (*left*), but not with AcrIE4-F7^{CTD} (*right*). The elution fractions were analyzed by SDS-PAGE. Uncropped gel images are shown in Figure S12. (B) ITC trace for the binding of AcrIE4-F7^{NTD} to Cas8e. The isotherm is representative of triplicate measurements and annotated with the average dissociation constant (K_D) and standard error.

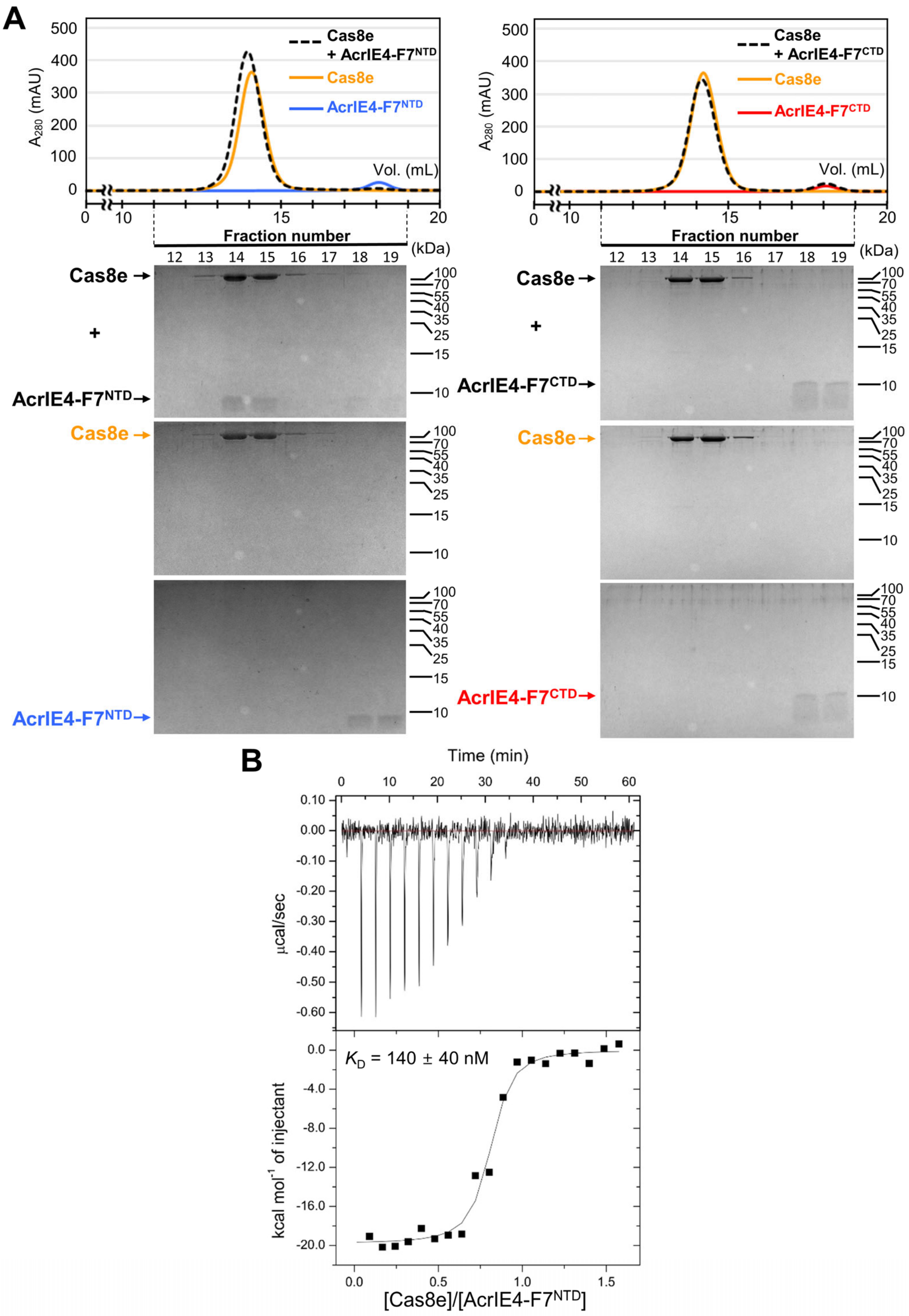

Figure S5. AcrIE4-F7 does not interact with *E. coli* Cas8e. In an analytical SEC analysis, AcrIE4-F7 did not co-elute with *E. coli* Cas8e. The elution fractions were analyzed by SDS-PAGE. Uncropped gel images are shown in Figure S12.

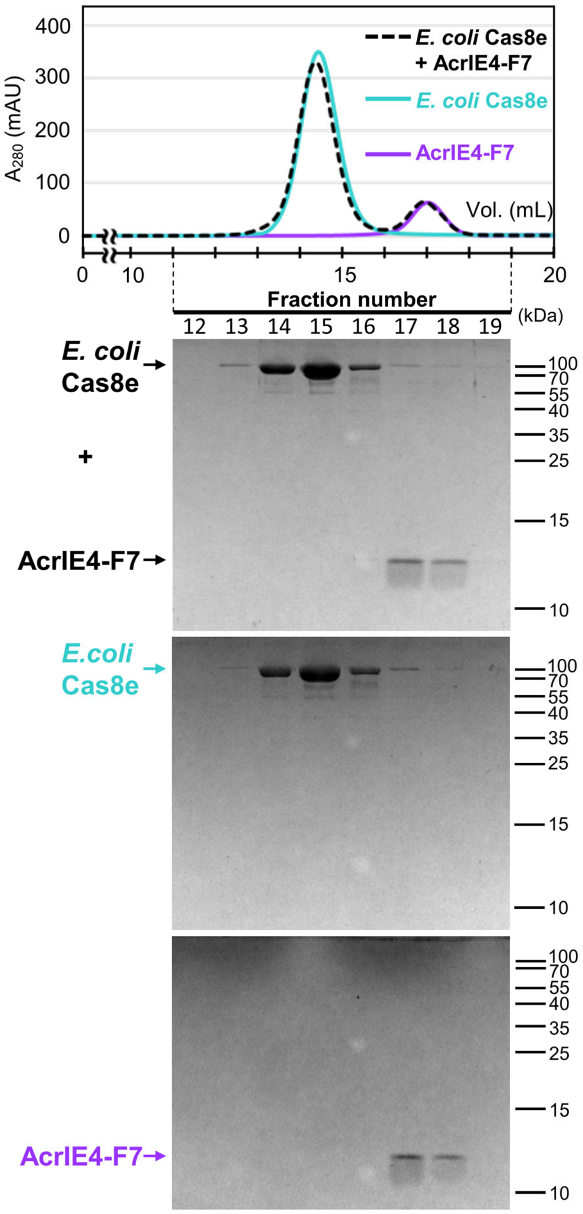

Figure S6. Sequence identity from pairwise protein sequence alignments of the *P. aeruginosa* and *E. coli* type I-E Cascade components.

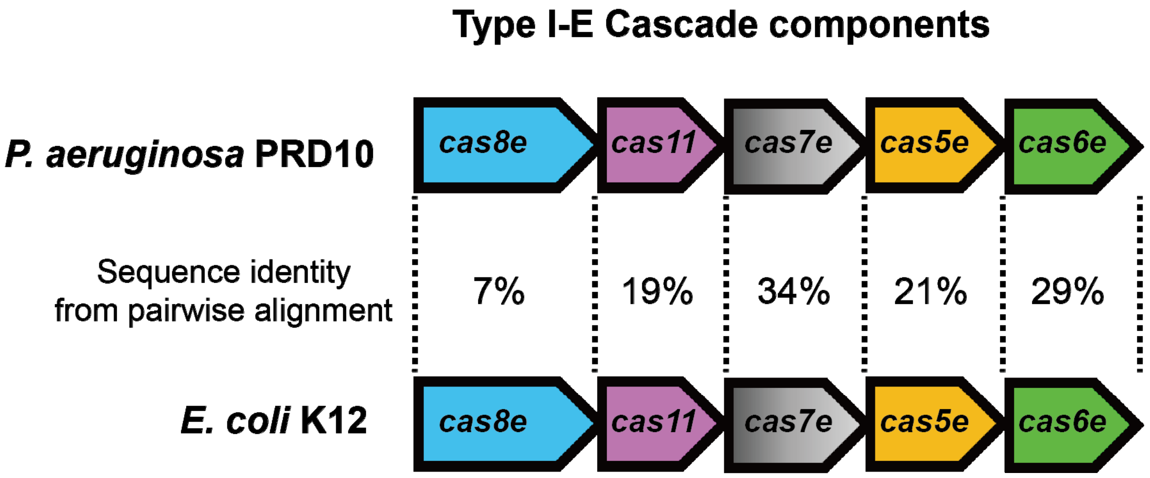

Figure S7. Structural comparison of the cryo-EM structure of *T. fusca* Cas8e (PDB code: 5U07) with a *P. aeruginosa* Cas8e model constructed with the Phyre2 program.

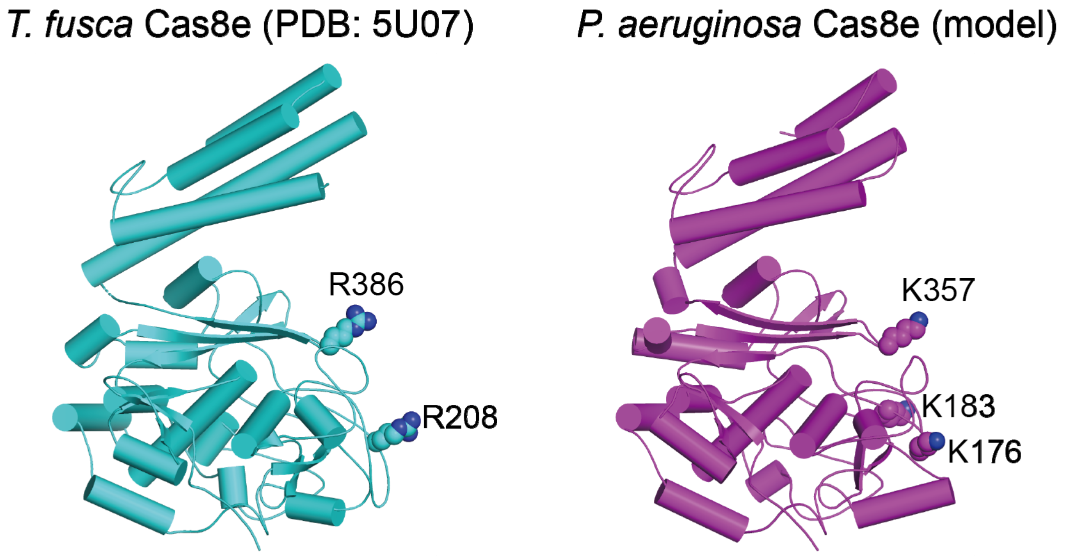

Figure S8. Cα trace model superposition of the solution structure of AcrIE4-F7^{CTD} (*red*) and the cryo-EM structure of native AcrIF7 (*purple*) complexed with the type I-F Cascade. The structures align well except for the β1–β2 loop that is flexible in solution.

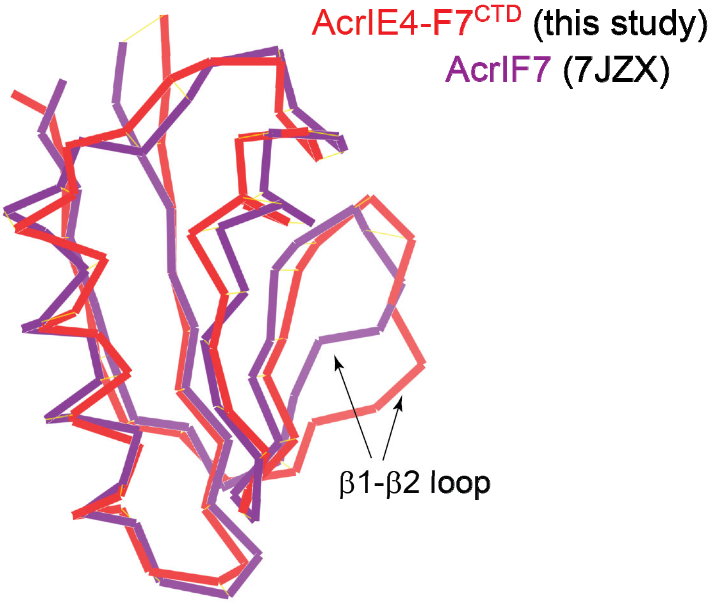

Figure S9. Analytical SEC for testing the interaction between Cas8e and the E19K/D22K mutant of AcrIE4-IF7. The mutant did not co-elute with Cas8e, demonstrating that the E19K/D22K mutations in AcrIE4-F7 completely abolished Cas8e binding. The SEC chromatogram for the interaction between Cas8e and WT AcrIE4-F7 (from Figure 1D) is indicated as a control with a dashed line for comparison. The elution fractions were analyzed by SDS-PAGE. Uncropped gel images are shown in Figure S12.

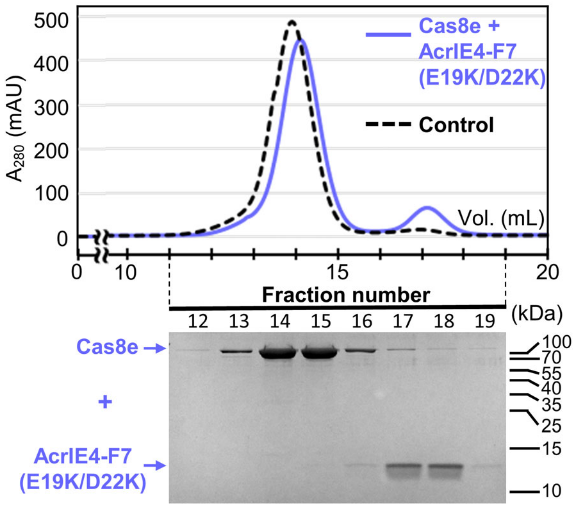

Figure S10. Integrated heats of injection for the interaction between Cas8e and various AcrIE4-F7 mutants, as calculated from ITC measurements. Titrations were carried out between Cas8e and the (A) E12K/D13K, (B) Y20A, and (C) E46K mutants. The isotherms are representative of triplicate measurements and annotated with their average dissociation constants (*K*D) and standard errors.

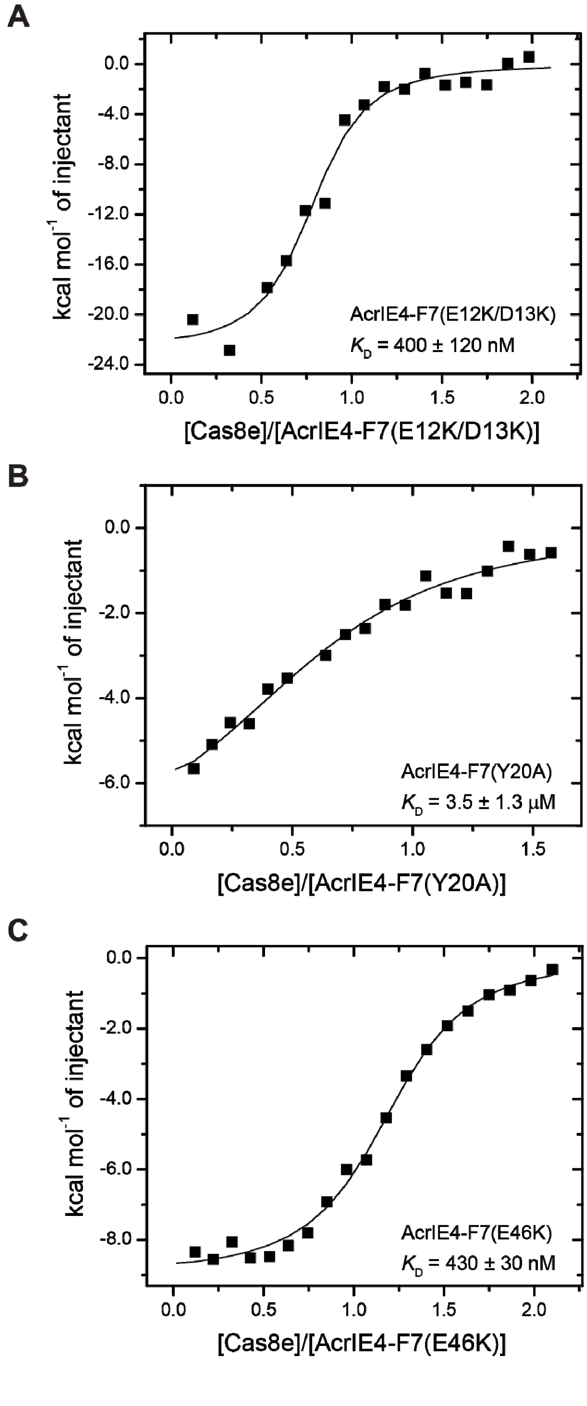

Figure S11. Circular dichroism spectra of WT and mutant AcrIE4-F7 measured in 10 mM sodium phosphate, pH 7.2 at 25°C using a J-815 circular dichroism spectropolarimeter. For visual clarity, only 5 CD spectra are shown per panel: WT AcrIE4-F7 and 4 mutants. (A) includes E12K/D13K, E19K/D22K, E19K, D22K. (B) includes Y20A, D30K/D31K, E38K/D39K, and E46K. All mutants exhibited CD spectra like the WT protein, indicating that their secondary structures remained intact.

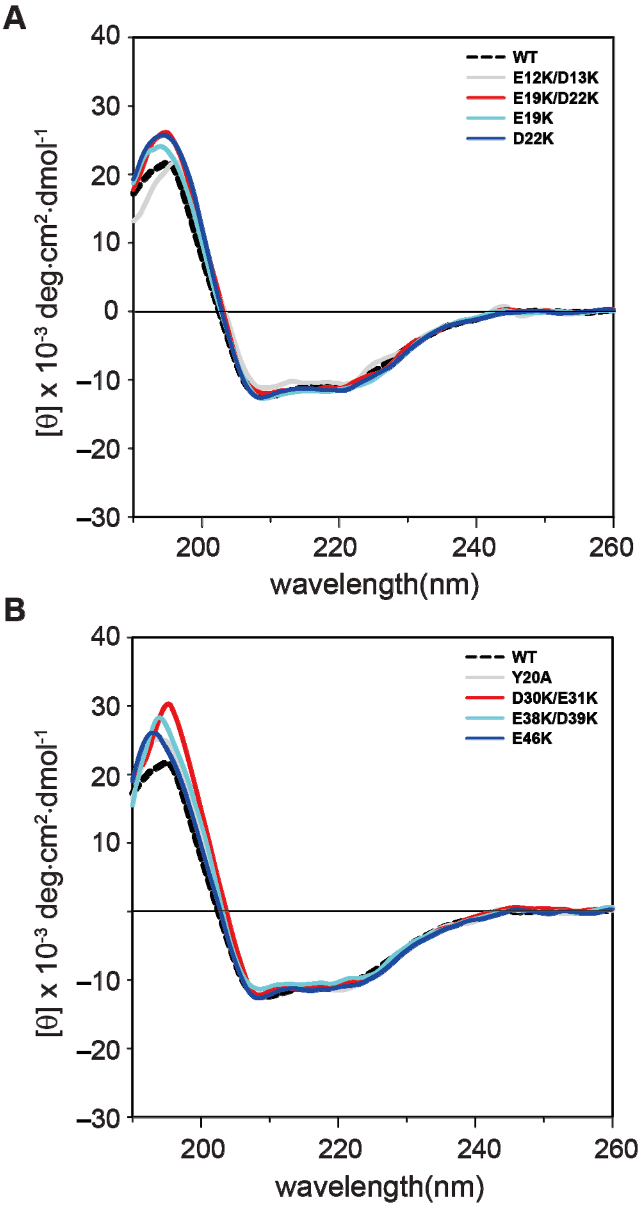

Figure S12. Uncropped gel images (Continued)

Fig. 1D: top

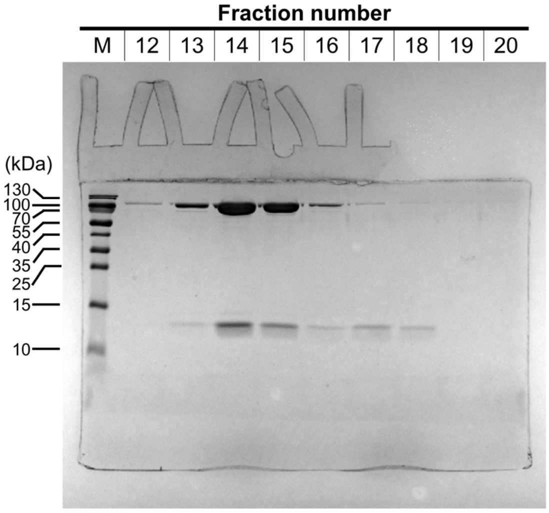

Fig. 2B: top

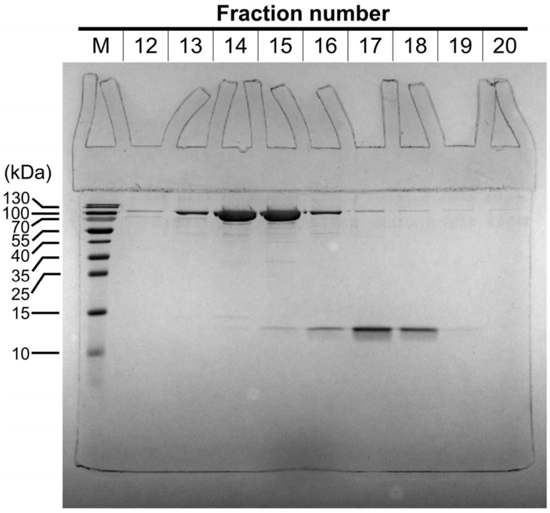

Fig. 1D: middle

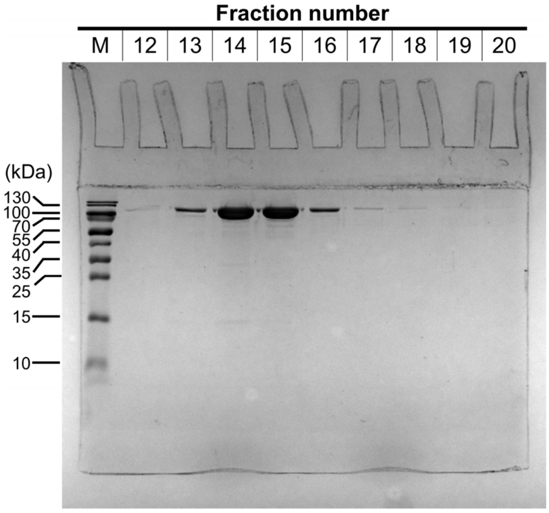

Fig. 2B: middle

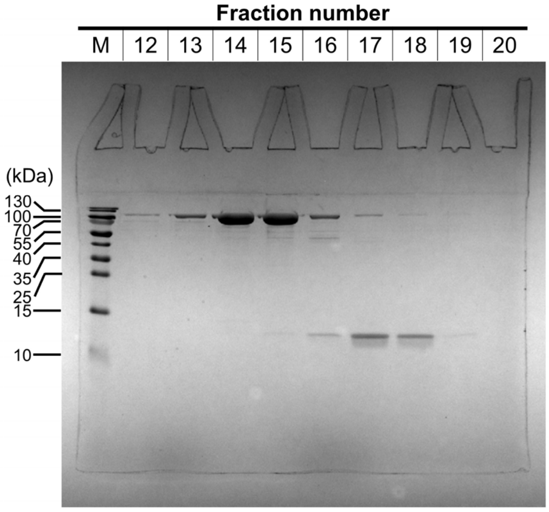

Fig. 1D: bottom

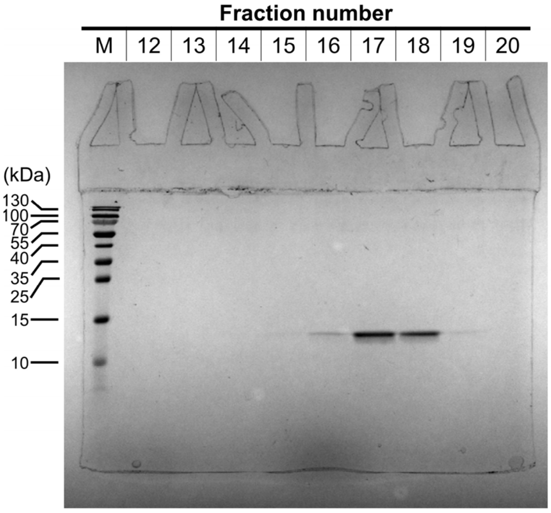

Fig. 2B: bottom

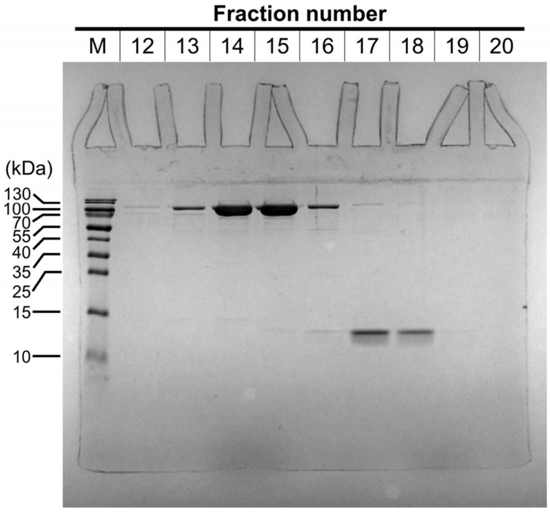

Figure S12. Uncropped gel images (Continued)

Fig. S1C: top

Fraction number

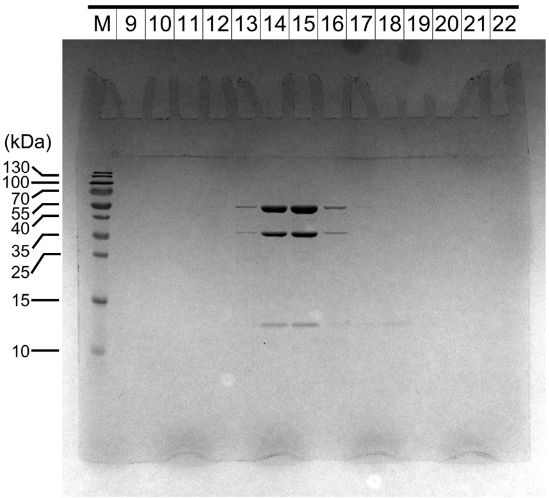

Fig. S2A: left, top

Fraction number

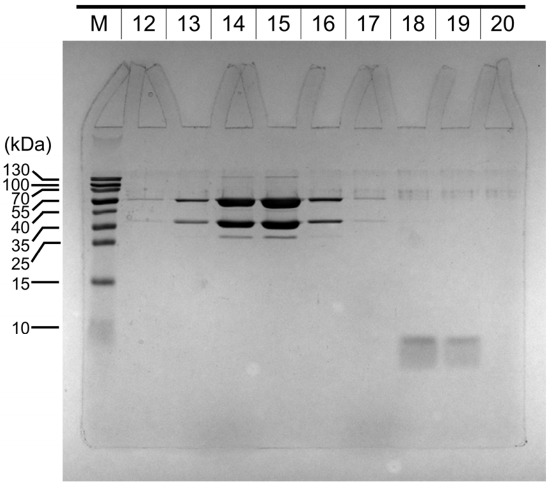

Fig. S1C: middle

Fraction number

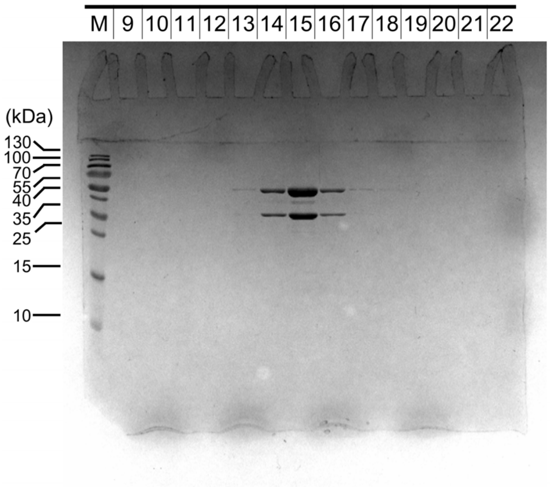

Fig. S2A: left, middle

Fraction number

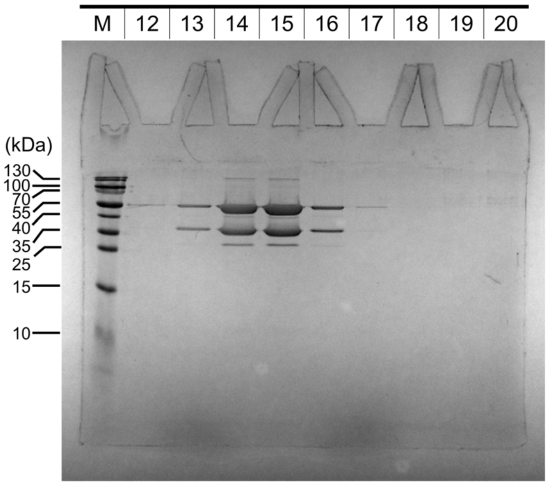

Fig. S1C: bottom

Fraction number

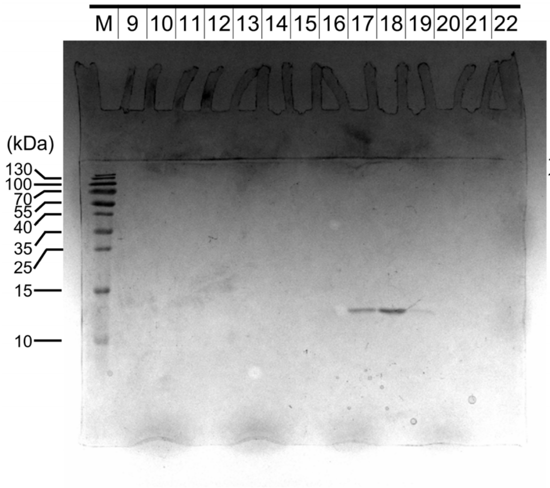

Fig. S2A: left, bottom

Fraction number

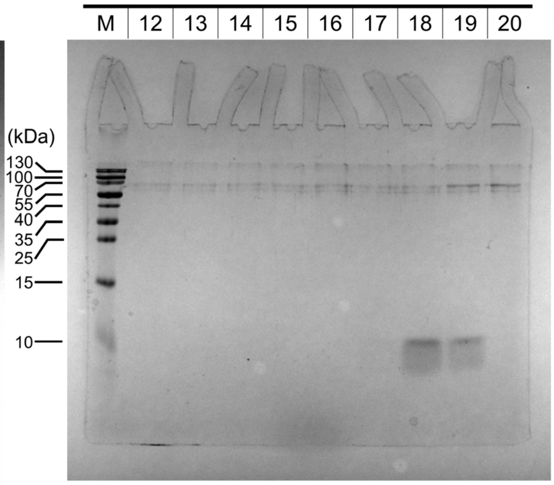

Figure S12. Uncropped gel images (Continued)

Fig. S2A: right, top

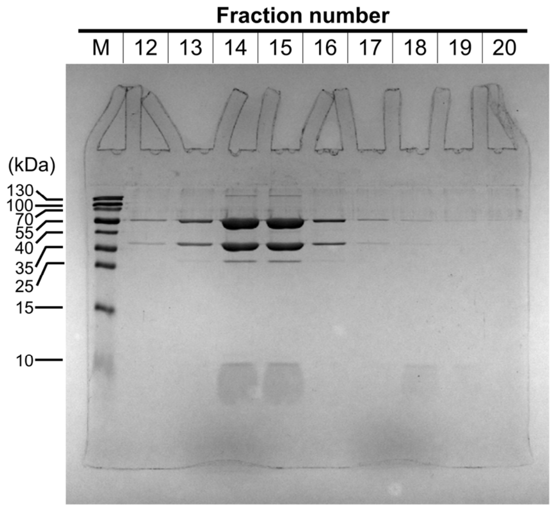

Fig. S3A: top

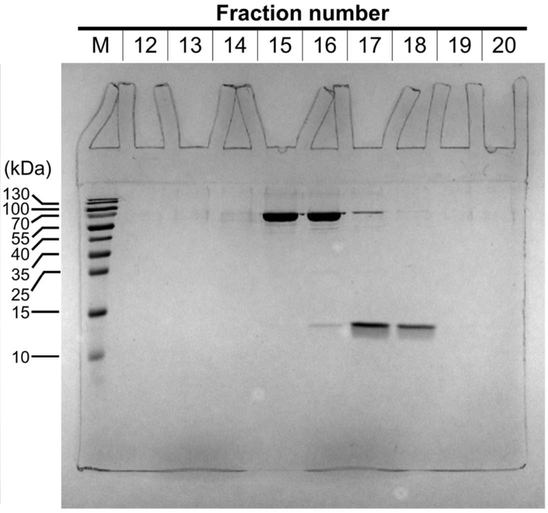

Fig. S2A: right, middle

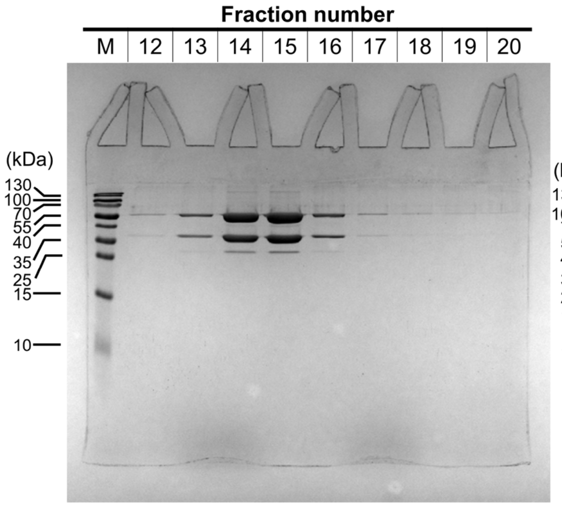

Fig. S3A: middle

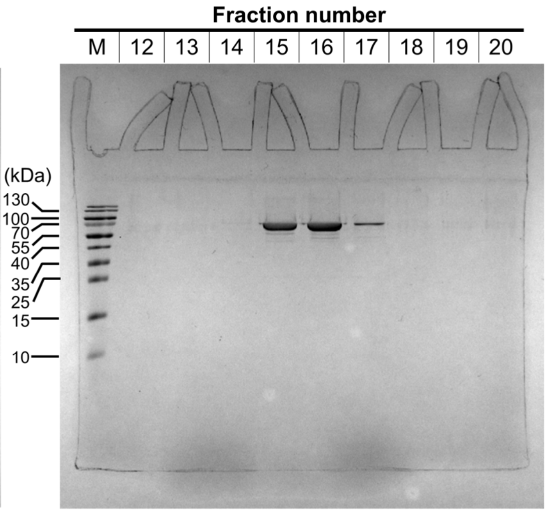

Fig. S2A: right, bottom

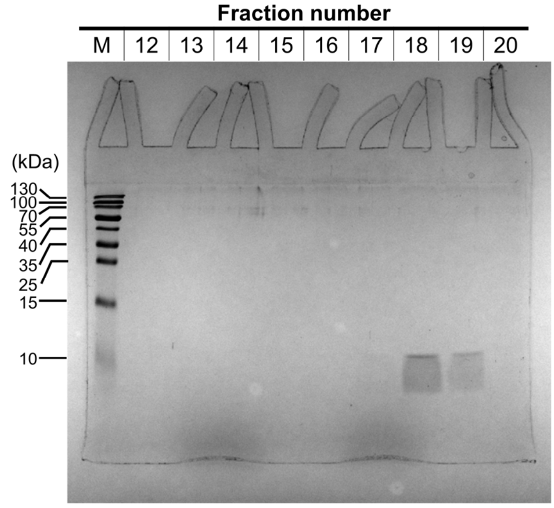

Fig. S3A: bottom

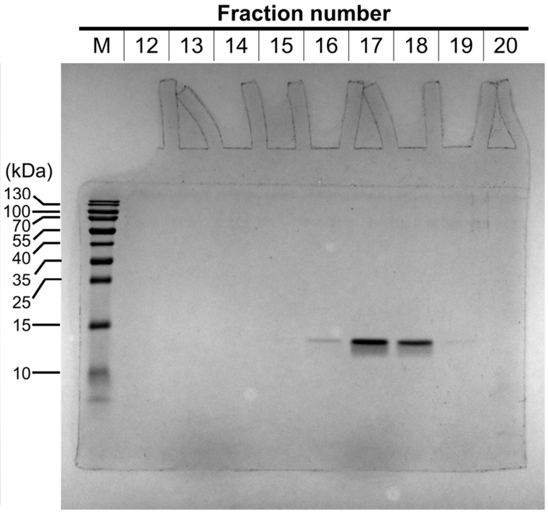

Figure S12. Uncropped gel images (Continued)

Fig. S3B: top

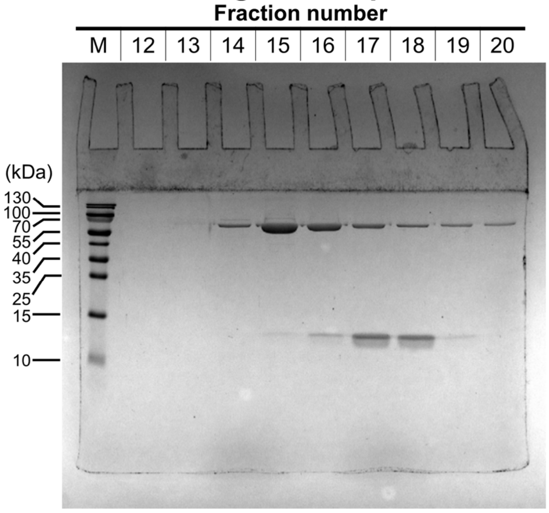

Fig. S3C: top

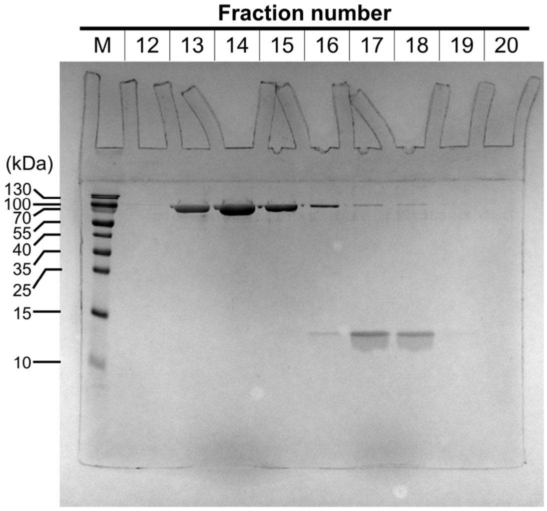

Fig. S3B: middle

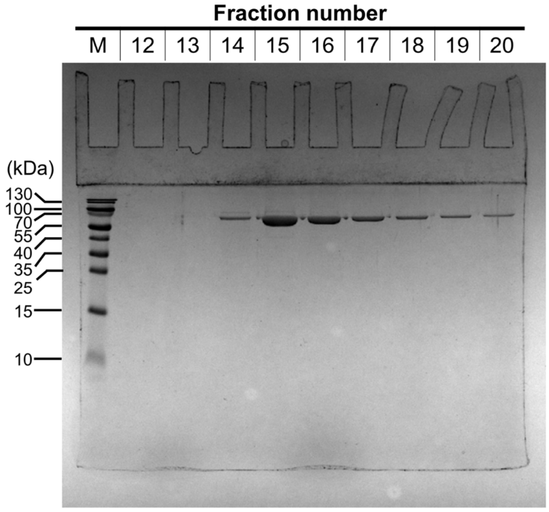

Fig. S3C: middle

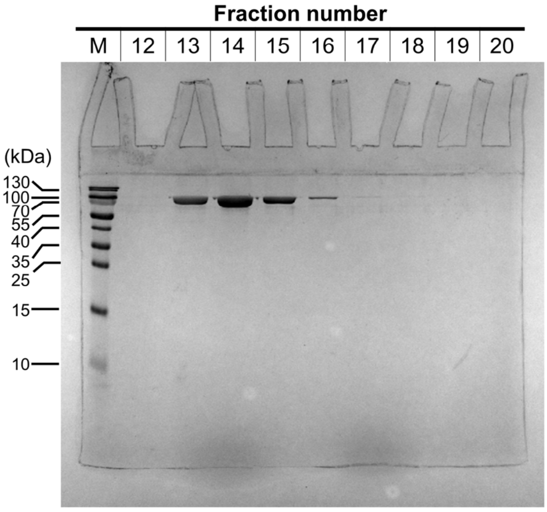

Fig. S3B: bottom

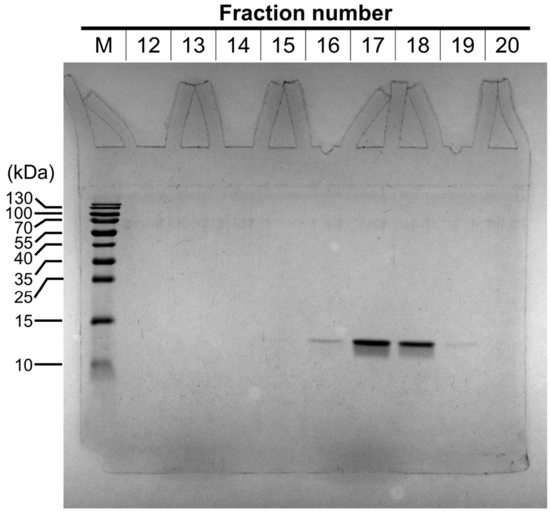

Fig. S3C: bottom

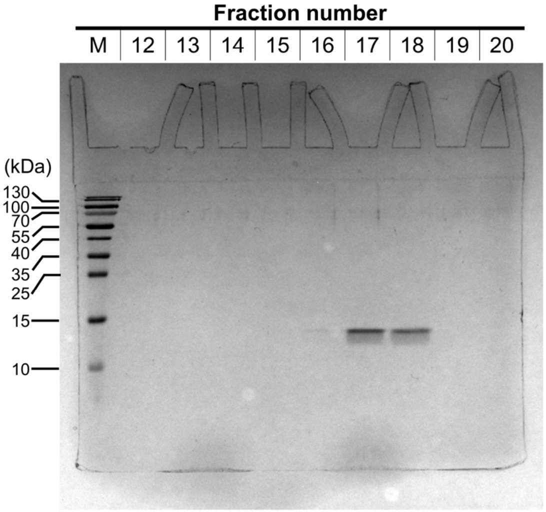

Figure S12. Uncropped gel images (Continued)

Fig. S3D: top

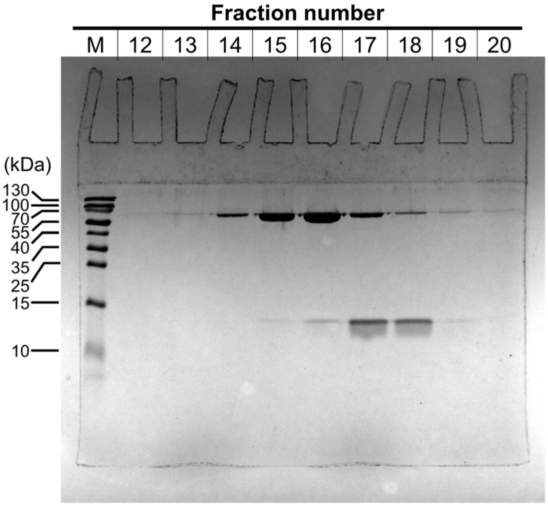

Fig. S4A: left, top

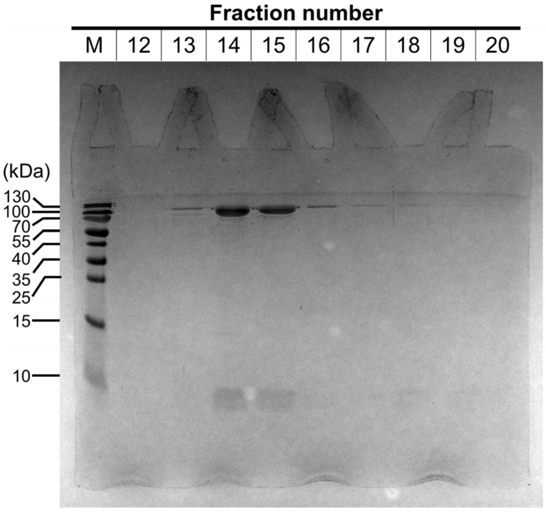

Fig. S3D: middle

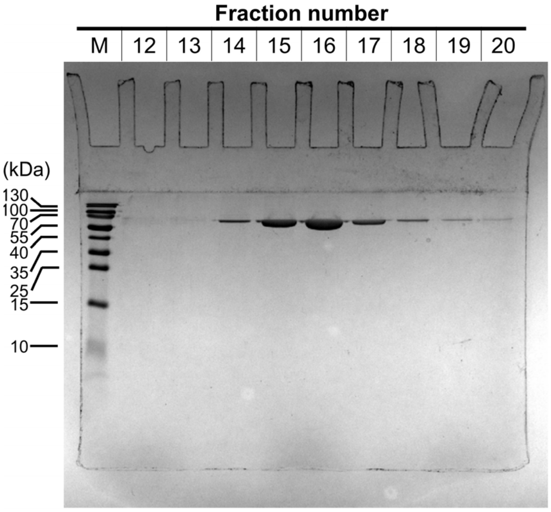

Fig. S4A: left, middle

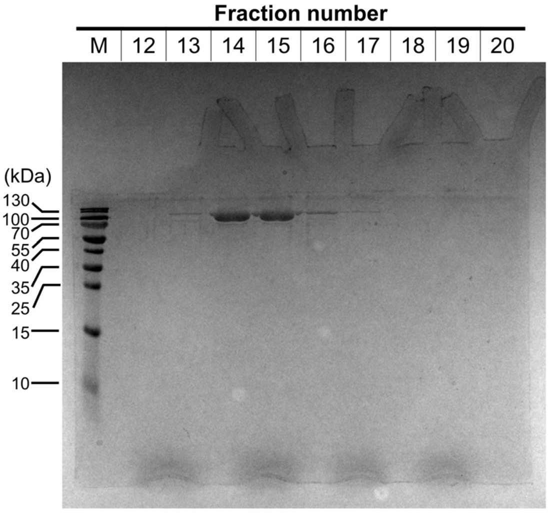

Fig. S3D: bottom

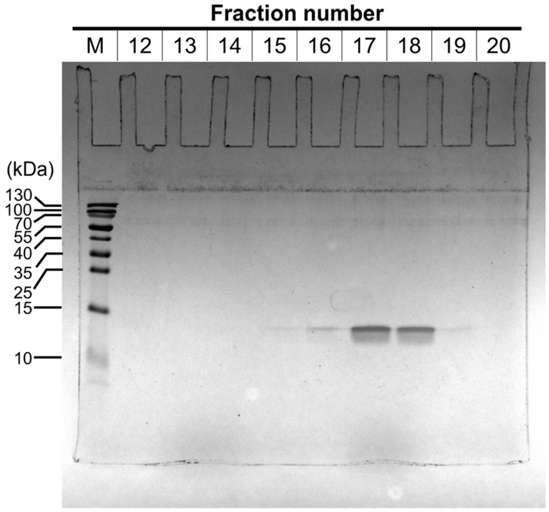

Fig. S4A: left, bottom

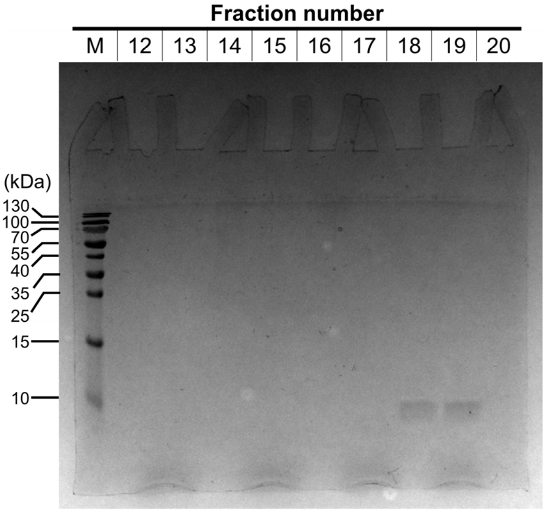

Figure S12. Uncropped gel images (Continued)

Fig. S4A: right, top

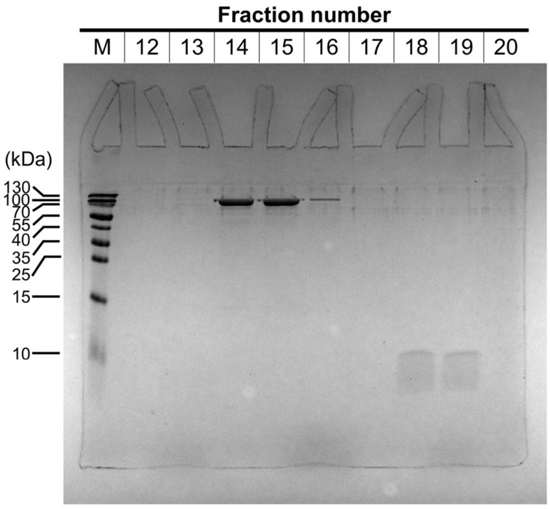

Fig. S5: top

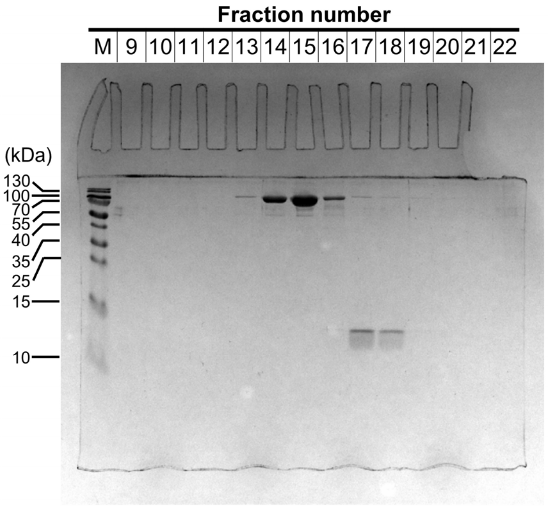

Fig. S4A: right, middle

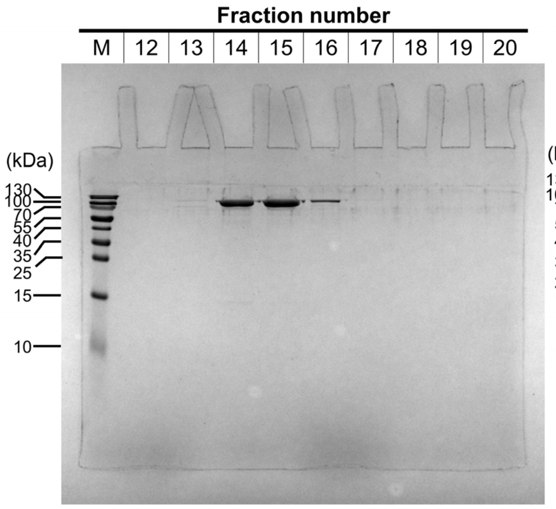

Fig. S5: middle

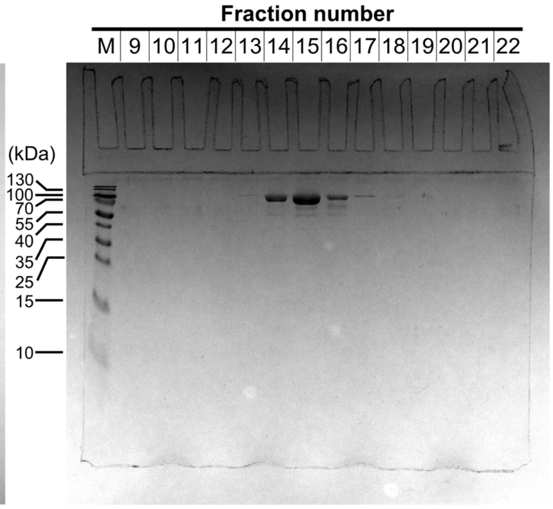

Fig. S4A: right, bottom

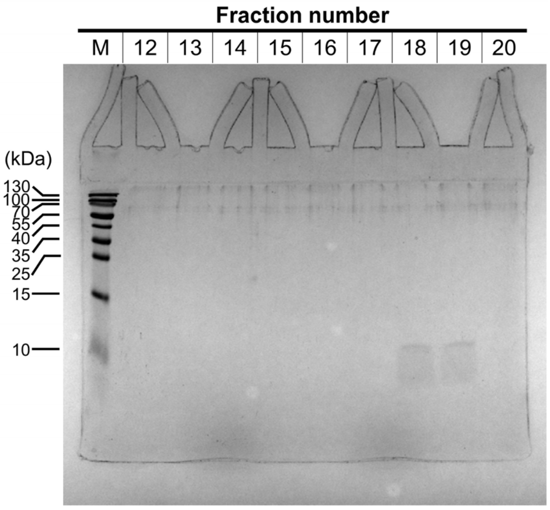

Fig. S5: bottom

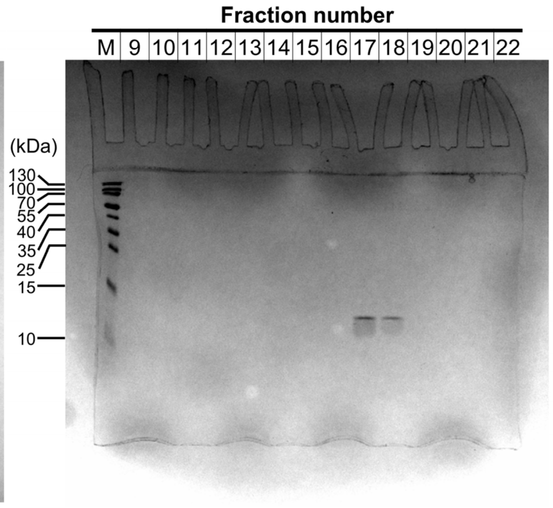

Figure S12. Uncropped gel images

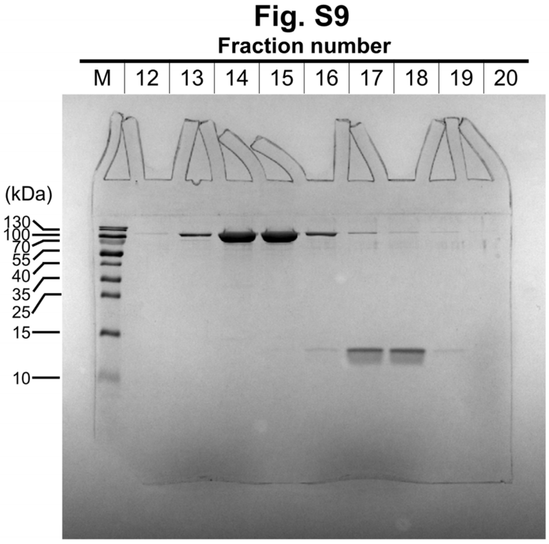

Supplement: gkac096_Supplemental_File [file gkac096_supplemental_file.pdf]
